# Aberrant STAT phosphorylation signaling in peripheral blood mononuclear cells from multiple sclerosis patients

**DOI:** 10.1186/s12974-018-1105-9

**Published:** 2018-03-07

**Authors:** Ester Canto, Noriko Isobe, Alessandro Didonna, MS-EPIC Study Group, S. Baranzini, C. Bevan, R. Bove, E. Crabtree-Hartman, J. M. Gelfand, D. S. Goodin, A. J. Green, R. Henry, J. Hollenbach, L. Kanaaneh, R. Lincoln, R. O’Shea, N. Papinutto, N. Ragan, G. Rush, W. A. Stern, S. S. Zamvil, Stephen L. Hauser, Jorge R. Oksenberg

**Affiliations:** Department of Neurology and Weill Institute for Neurosciences, University of California at San Francisco, Nelson Rising Lane, San Francisco, CA 94158 USA

**Keywords:** STAT proteins, Multiple sclerosis, Phosphoflow cytometry, Interferon signaling

## Abstract

**Background:**

Multiple sclerosis (MS) is characterized by increased activation of peripheral blood mononuclear cells (PBMCs), linked to perturbations in the phosphorylation of signaling proteins.

**Methods:**

We developed a phosphoflow cytometry protocol to assess the levels of 11 phosphorylated nuclear proteins at baseline conditions and after cell activation in distinct PBMC populations from 41 treatment-naïve relapsing-remitting (RR) MS subjects and 37 healthy controls, and in a second cohort of 9 untreated RRMS patients and 10 secondary progressive (SP) MS patients. Levels of HLA-ABC, HLA-E, and HLA-DR were also assessed. Phosphorylation levels of selected proteins were also assessed in mouse splenocytes isolated from myelin oligodendrocyte glycoprotein (MOG)_35–55_-induced experimental autoimmune encephalomyelitis (EAE).

**Results:**

Modest differences were observed at baseline between patients and controls, with general lower phosphorylation levels in cells from affected individuals. Conversely, a dramatic increase in phosphorylated p38MAPK and STAT proteins was observed across all cell types in MS patients compared to controls after in vitro activation. A similar phosphorylation profile was observed in mouse lymphocytes primed in vivo with MOG. Furthermore, levels of all p-STAT proteins were found directly correlated with HLA expression in monocytes. Levels of phosphorylated proteins did not differ between relapsing-remitting and secondary progressive MS patients either in baseline conditions or after stimulation. Lastly, phosphorylation levels appear to be independent of the genotype.

**Conclusion:**

The response to IFN-α through STAT proteins signaling is strongly dysregulated in MS patients irrespective of disease stage. These findings suggest that the aberrant activation of this pathway could lead to changes in the expression of HLA molecules in antigen presenting cells, which are known to play important roles in the regulation of the immune response in health and disease.

**Electronic supplementary material:**

The online version of this article (10.1186/s12974-018-1105-9) contains supplementary material, which is available to authorized users.

## Background

The primary pathogenic role of inflammatory responses in multiple sclerosis (MS) and its broadly used animal model, experimental autoimmune encephalomyelitis (EAE), is supported by a large body of laboratory, pathology, and genetic data [1–4]. Furthermore, clinical-based evidence from more than 20 years of immunotherapy firmly confirms that this neurodegenerative disease is at its core autoimmune. However, the early mechanisms driving the immune dysregulation in the periphery that lead to central nervous system (CNS) myelin breakdown, axonal damage, and neuronal cell death, are not fully understood. Intriguingly, genetically determined changes in signaling responses and activation thresholds in specific immune cell lineages have been described in MS [5]. Identifying dysregulated signaling pathways in cell compartments relevant to the disease and their relationship with genetic variance could represent a useful strategy to better understand the natural history of MS and discover new therapeutic targets.

Perturbations of cell-specific signaling pathways can be efficiently assessed using a flow cytometry-based technique known as phosphoflow cytometry [6–8]. Phosphoflow cytometry measures the activation status of key intracellular signaling molecules in response to different stimuli (e.g., antigen recognition through antigen receptors, unspecific stimulations, or activation of cytokine receptors), through the quantification of the phosphorylated forms of transcription factors or tyrosine and serine threonine kinases, which in turn are responsible for the downstream phosphorylation of multiple intracellular targets (reviewed in [7]). Phosphoflow cytometry has been used in the past few years to assess the activation status of cells in various autoimmune diseases such as systemic lupus erythematosus and rheumatoid arthritis [9, 10], in which changes in protein phosphorylation have been associated with disease activity.

Abnormal levels of phosphorylated proteins belonging to the signal transducers and activators of transcription (STAT) family in the peripheral blood mononuclear cells (PBMCs) of MS patients have been associated with a number of clinically relevant phenotypes, including the development of clinically definite MS in individuals presenting a clinically isolated syndrome (CIS) [11], the development of flares [12], and high titers of neutralizing antibodies against interferon beta (IFN-β) [12–14]. Noteworthy, inhibitors of the JAK/STAT pathway have shown to ameliorate the clinical signs of EAE by means of inhibiting CD4 T cell differentiation toward the pro-inflammatory Th1/Th17 subtypes [15, 16]. The analysis of both baseline and activated levels of phosphoproteins can provide therefore, important information to identify disease-associated biochemical pathways that are either abnormally upregulated or downregulated. However, there are no studies investigating the differences in the phosphorylation response to in vitro activators between MS patients and healthy controls. In the present study, we applied phosphoflow cytometry to simultaneously measure for the first time the induced phosphorylation response of 11 different proteins in five peripheral blood immune cell subsets in treatment naïve, short disease duration MS patients and matched controls. The results show that PBMCs from MS patients have an innate propensity to heighten protein phosphorylation in response to stimuli, in particular STAT proteins, which are found consistently upregulated upon stimulation across all the analyzed cell subsets of the blood.

## Methods

### Subjects

The study included 60 MS patients enrolled and evaluated at the Multiple Sclerosis Center, University of California, San Francisco (UCSF), participating in a prospective observational cohort [17]. Analysis was organized in two groups; the first consisted of 41 relapsing-remitting (RR) untreated MS patients and 37 gender- and age-matched healthy controls, and the second consisted of 9 untreated RRMS patients and 10 secondary progressive (SP) MS patients matched by gender, age, and disease duration. Inclusion and exclusion criteria were previously described, but patients fulfilled the International Panel on Diagnostic criteria for MS [18] and were untreated at the time of blood sampling.

In addition to extensive clinical data, 4-digit resolution HLA allele typing for *HLA-A*, *HLA-B*, *HLA-C*, *HLA-DRB1*, and *HLA-DQB1* was available for all study participants. Genome-wide single nucleotide polymorphisms (SNPs) genotypes were available for 32 MS study participants. Using the most updated list of established non-MHC MS-associated variants (*n* = 200) [19], we calculated the overall cumulative genetic burden (MSGB) and two specific ontological pathway genetic burdens for each study participant: MSPBphos (risk SNPs in prioritized genes in the protein phosphorylation ontological family) and MSPBregphos (risk SNPs in prioritized genes in the regulation of protein phosphorylation ontological family). Each polygenic score was determined as the allele dose multiplied by the reported allele effect size [19–21]. In addition to MSGB scores, we also selected single STAT-associated SNPs to assess individual effects of specific variants on the regulation of phosphorylation responses. For that purpose, we limited the search to validated MS risk variants [19] located within 1 Mb from the STAT protein genes. Three SNPs in the proximity of STAT genes: rs6738544 (close to STAT1 and STAT4 genes), rs1026916 (close to STAT3 and STAT5 genes), and rs701006 (close to STAT6 gene), were identified and selected for the analysis of association with levels of phosphorylation of STAT proteins. Genotyping data for STAT1/4 SNP was available for 32 MS patients and 20 controls and genotyping for STAT3/5 and STAT6 SNPs was available for 32 patients.

The Committee on Human Research at UCSF approved the study protocol. Written informed consent was obtained from all the participants. The clinical and demographic characteristics of the patients and controls are shown in Table 1.

**Table 1:** Clinical characteristics of patients and controls

	RRMS vs controls			RRMS vs SPMS		
Clinical variable	Controls *n* = 37	RRMS *n* = 41	*p* value	RRMS *n* = 9	SPMS *n* = 10	*p* value
Gender (female/male)	28/9	31/10	1.0	9/0	10/0	1.0
Age (years)	42.9 (10.1)	43.5 (9.6)	0.932	57.4 (8.8)	56.3 (9.6)	0.870
Disease duration (years)	–	4.6 (2.6)	–	23.5 (6.3)	22.7 (6.5)	0.806
EDSS	–	1.8 (1.1)	–	2.6 (1.2)	5.0 (1.5)	3.4 × 10^− 3^

Clinical characteristics of the patients and controls used for the study. Values shown as mean (standard deviation). *RRMS* relapsing-remitting multiple sclerosis, *SPMS* secondary progressive multiple sclerosis

### Phosphoflow cytometry and HLA assessment by flow cytometry

We adapted and modified a previously reported protocol [8] to simultaneously determine baseline and activated levels of 11 phosphoproteins (Bruton’s tyrosine kinase (Btk), Phosphoinositide phospholipase C (PLCγ), Protein kinase B (Akt), Casitas B-lineage Lymphoma (Cbl), P38 mitogen-activated protein kinase (p38MAPK), Extracellular Signal-regulated Kinase 1/2 (Erk1/2) and Signal Transducers and Activators of Transcription 1, 3, 4, 5, 6 (STAT1, STAT3, STAT4, STAT5, STAT6)) by flow cytometry in five different peripheral blood cell types (CD4 T cells, CD8 T cells, B cells, monocytes, and NK cells). Briefly, 10 million cryopreserved PBMCs were thawed and allowed to rest for 1 h at 37 °C. Then, cells were stimulated for 15 min with H_2_O_2_ (45 mM) for Btk, PLCγ, Akt, and c-Cbl activation in response to oxidative stress, Phorbol 12-myristate 13-acetate (PMA) (400 nM; Sigma-Aldrich) for p38MAPK and Erk1/2 activation, and human IFN-α (40,000 U/mL; Millipore) or recombinant human IFN-γ (4000 U/mL; R&D Systems) for STAT proteins activation. For baseline levels assessment, cells were left unstimulated for 15 min at 37 °C. Cells were subsequently fixed with formaldehyde (final concentration 1.5%) and permeabilized with 100% ice-cold methanol for 30 min on ice. After washing, cells were stained for the phosphoproteins and cell-specific markers. A representative dot plot showing the gating strategy for the analysis of the different blood cell populations and the antibodies used for staining is detailed in Additional file 1: Figure S1. Fold changes of protein phosphorylation were calculated as the ratio of the mean fluorescence intensity (MFI) after stimulation over the MFI in baseline conditions.

In a subgroup of 21 patients and 21 matched healthy controls, levels of total p38MAPK, Erk1/2, STAT1, and STAT6 were assessed following the same protocol as described above without adding any stimuli to the cells. The antibodies used for staining are detailed in Additional file 1: Figure S1.

For the analysis of HLA-E, HLA-ABC, and HLA-DR expression, cells were stimulated with either human IFN-α (40,000 U/mL; Millipore), recombinant human IFN-γ (4,000 U/mL; R&D Systems) or were left unstimulated for 24 h at 37 °C. Then, cells in suspension were collected and the adherent cells were detached with PBS-0.05% EDTA on ice for 30 min. Cells were then washed with PBS, blocked with Fc blocking reagent (Miltenyi Biotech, San Diego) following the manufacturer’s protocol, and subsequently stained with cell-specific marker antibodies and HLA-E, HLA-ABC, and HLA-DR antibodies.

### EAE induction and antigen-specific phosphoflow

Five 8-weeks-old C57BL/6 J female mice (from Jackson Laboratories) were injected subcutaneously with 100 μg MOG_35–55_ (EZBiolab), in complete Freund’s adjuvant (DIFCO Laboratories). Mice received 400 ng of pertussis toxin by intraperitoneal injection at day 0 and at day 2. Five control mice were mock-injected with everything except for the MOG peptide. All animal experiments were conducted according to protocols approved by the local animal welfare committee. Spleens from EAE and control mice were harvested 15 days post immunization (dpi) and splenocytes were isolated. Cells were stimulated with MOG_35–55_ (10 μg/mL, EZBiolab) for 24 h or left unstimulated. Then, splenocytes were fixed with formaldehyde (final concentration 1.5%) and permeabilized with 100% ice-cold methanol for 30 min on ice. After washing, cells were stained for the phosphoproteins and cell markers with the antibodies in Additional file 1: Figure S1.

### Statistical analysis

A Wilcoxon test was used to compare phosphorylation status between cases and controls, and *t* test was used to compare phosphorylation levels between EAE and control mice. Spearman correlation coefficients were calculated to study correlations between variables. *p* values less than 0.05 were considered significant. All statistical analyses were computed using code written in R software (r-project.org) 3.3.1 version. In order to minimize batch effects, samples from both patients and controls were normalized using the Generalized Feature Scaling (GFS) method. To generate heatmaps, MFI values or fold change values for each phosphorylated protein in each cell type were transformed into *z*-scores and plotted according to the following color code: samples with levels of phosphorylated proteins higher than the mean are depicted in red while proteins showing levels of phosphorylated proteins lower than the mean are depicted in blue.

## Results

### Peripheral blood mononuclear cells from MS patients are highly sensitive to in vitro activation

**Fig. 1:**
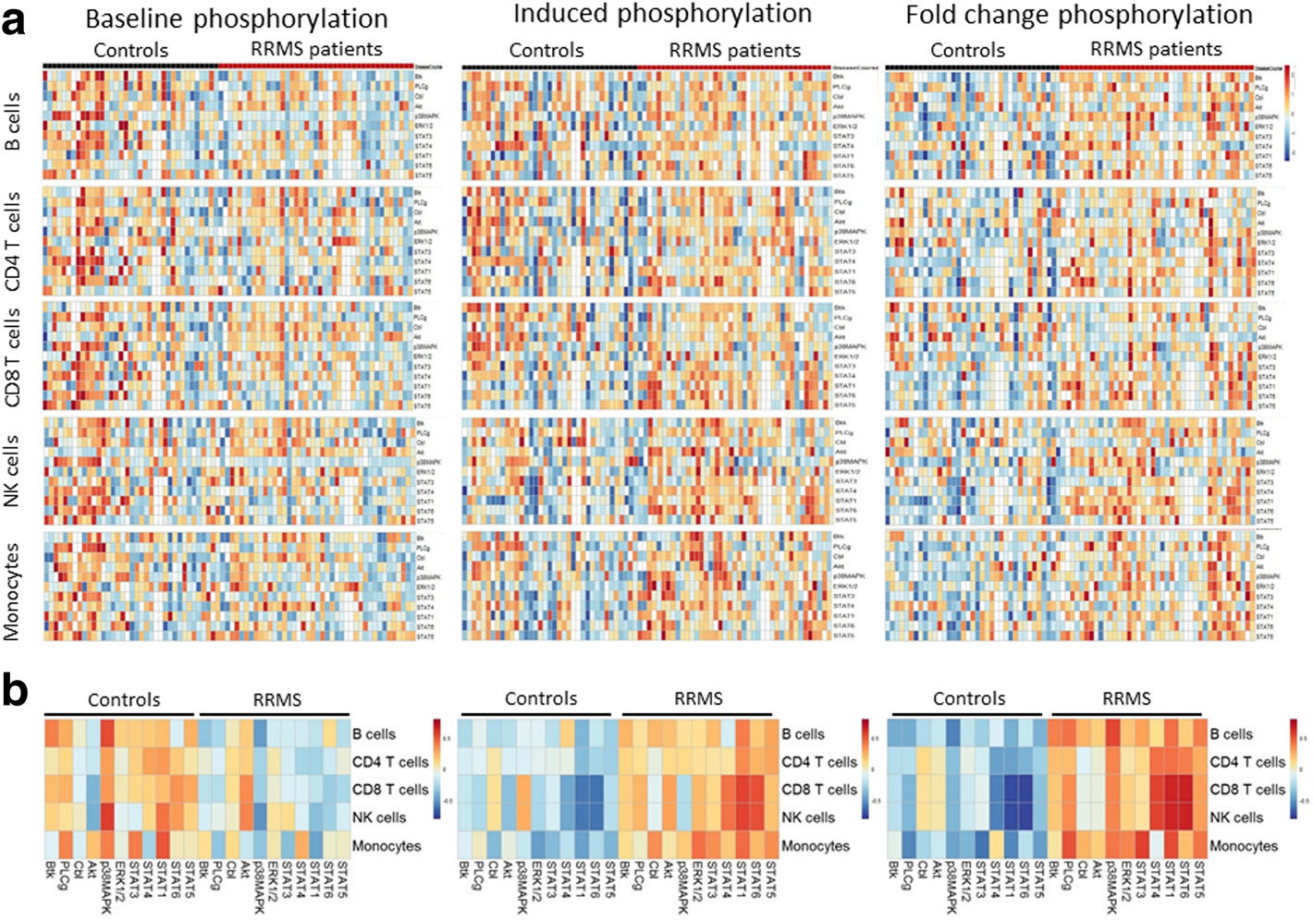
Immune cells from MS patients show a distinct phosphorylation signature in baseline conditions and upon in vitro stimulation. PBMCs from RRMS patients (*n* = 41) and healthy controls (*n* = 37) were either left unstimulated or activated for 15 min with H_2_O_2_ for Btk, PLCγ, Akt, and c-Cbl activation, PMA for p38MAPK and Erk1/2 activation, and human IFN-α for STAT proteins activation. **a** The individual *z*-score transformation of the MFI of each phosphorylated protein or the fold change of phosphorylation has been represented for each cell population in each subject of the study. **b** The summary of the *z*-scores of the MFI of each phosphorylated protein or the fold change of phosphorylation has been represented for each cell population in MS patients and controls. MS patients show lower levels of phosphorylated proteins in baseline conditions, and increased levels of phosphorylated proteins and increased fold changes of phosphorylation after stimulation compared to healthy controls. Levels of phosphorylated proteins higher than the mean are shown in red and levels of phosphorylated proteins lower than the mean are shown in blue

The most consistent difference observed between patients and controls in baseline conditions was a generalized decrease in levels of the tested phosphorylated proteins. In particular, the levels of phosphorylated p38MAPK were found significantly decreased in MS patients across all cell types (Fig. 1, Additional file 2: Table S1). STAT1 phosphorylation levels were also found decreased in CD4 T cells (*p* = 0.014), NK cells (*p* = 0.002) and monocytes (*p* = 2.05 × 10^− 4^). Furthermore, levels of phosphorylated STAT6 (*p* = 0.018, *p* = 0.048) were decreased, while phosphorylated Akt was found increased in CD8 T cells (*p* = 0.003) and NK cells (*p* = 0.001) from MS patients compared to controls.

The different in vitro stimuli induced protein phosphorylation in all the analyzed cell types, both in patients and healthy controls (Additional file 3: Table S2). However, the phosphorylation fold change in response to the different stimuli was found increased in patients with MS compared to controls. Specifically, the response to oxidative stress was found significantly increased in patients’ samples for p-Cbl (*p* = 0.007) and p-Akt (*p* = 0.017) in monocytes and CD8 T cells, respectively (Fig. 1, Additional file 3: Table S2). Likewise, a significant increment in p-Erk1/2 (*p* = 0.003) in monocytes and in Btk (*p* = 0.001) in B cells was observed. Interestingly, a fold change increase in phosphorylated p-p38MAPK was observed in all cell types except CD8 T cells. Additionally, we observed a consistent increase in phosphorylation of STAT proteins in response to IFN-α in all cell types, but especially strong in the NK cell population (*p* value range 0.006 to 2.04 × 10^− 10^). Finally, the absolute levels of phosphorylated STAT proteins were found to have increased after stimulation in MS patients compared to controls (Fig. 1, Additional file 4: Table S3).

In order to rule out the possibility that the observed differences in the levels of phosphorylated proteins and phosphorylation response were caused by differences in the absolute levels of those proteins, we measured the total levels of p38MAPK, Erk1/2, STAT1, and STAT6 (the ones that showed the most significant differences between patients and controls) in a subgroup of RRMS patients and controls. Age, disease duration, and EDSS in this subset were similar to the full dataset (*p* = 0.709, *p* = 0.294, and *p* = 0.871, respectively). We found no differences in the total levels of these proteins between MS patients and controls (Additional file 5: Table S4).

### Phosphorylation response and clinical variables

Protein phosphorylation has been previously associated with disease activity or severity in other autoimmune diseases; hence, we were interested in exploring the correlation between levels of protein phosphorylation after stimulation and the Expanded Disability Status Scale (EDSS), which is a broadly used measure of disease disability in MS. We found a weak correlation between levels of p-Erk1/2 in CD4 T cells (Spearman corr = 0.55; *p* = 2.9 × 10^− 03^) and CD8 T cells (spearman corr = 0.48; *p* = 1.4 × 10^− 02^) and EDSS, but not for any of the other phosphoproteins analyzed. Interestingly, we observed that activated levels of p-STAT3 and p-STAT5 in NK cells from MS subjects positively correlate with disease duration (*r* = 0.34, *p* = 0.035 and *r* = 0.43, *p* = 0.0075) but not with age (*r* = − 0.06, *p* = 0.73 and *r* = 0.06, *p* = 0.73), suggesting that changes in the susceptibility to STAT phosphorylation might reflect a physiological mechanism associated with the disease, independent of aging. Next, we determined the levels of phosphorylated proteins in a cohort of untreated patients who had transitioned to SPMS (*n* = 10) compared to a group with similar disease duration and age that did not (*n* = 9). No differences were detected either in baseline conditions or after stimulation (Additional file 6: Table S5 and Additional file 7: Table S6).

### Phosphorylation levels of STAT proteins and MS risk genomic variants

We next assessed whether the increased phosphorylation in STAT proteins observed in MS patients in response to stimuli was related to the 200 genetic variants associated with MS susceptibility. We focused on STAT proteins because of their consistent increase of phosphorylation observed across all cell types. For this purpose, we first considered the correlation between levels of all phosphorylated proteins and the MSGB, MSPBphos, or MSPBregphos, which are weighted cumulative scores that summarize the total or ontological polygenic genetic burden of MS risk variants for each individual. No consistent correlations between these variables and protein phosphorylation were seen (Additional file 8: Table S7 and Additional file 9: Table S8). Likewise, when we assessed the correlation between p-STAT proteins and individual risk SNPs located within 1 Mb from the STAT protein genes, we did not observe a difference in levels of p-STAT proteins between the different genotypes that could help explain the increase observed in MS. In addition, no differences in phosphorylated protein levels were observed between individuals carrying 0, 1, or 2 copies of the HLA risk allele HLA-DRB1*15:01 (data not shown). Overall, these results suggest that the increased STAT protein phosphorylation observed in MS patients might not be affected by the disease risk loci, although this lack of association might be due to the limited power in our dataset. Thus, larger studies will be required to firmly prove or disprove an association between variants affecting the risk of developing MS and the dysregulated signaling pathways.

### Phosphorylation of STAT proteins correlates with HLA expression after IFN-α and IFN-γ stimulation in monocytes

Previous studies have shown that interferons modulate the expression of major histocompatibility complex (MHC) class I and MHC class II molecules in T cells and monocytes, as well as in other cell types such as tumor cell lines [22, 23]. This process is mediated by STAT proteins [24]. Hence, we were interested in assessing if there was an association between STAT protein phosphorylation levels and the expression of classical HLA-A, B, C, and DR and non-classical HLA-E both at baseline and after stimulation with either IFN-α or IFN-γ. First, we observed that IFN-α induces class I HLA-E and HLA-ABC expression in all cell types independent of disease status, while it mainly induces class II HLA-DR expression in NK cells and in monocytes (Table 2). Similarly, stimulation with IFN-γ induces modest increases of HLA-E and HLA-ABC expression in all cell types, but a very strong upregulation of HLA-DR in monocytes and to a lesser degree in NK cells.

**Table 2:** Fold change of HLA expression induced by IFN-α and IFN-γ in each cell type analyzed

	Fold change HLA-E (IFN-α)	Fold change HLA-E (IFN-γ)	Fold change HLA-ABC (IFN-α)	Fold change HLA-ABC (IFN-γ)	Fold change HLA-DR (IFN-α)	Fold change HLA-DR (IFN-γ)
B cells	1.733	1.503	1.286	1.202	0.979	1.041
CD4 T cells	1.790	1.514	1.554	1.267	0.921	1.070
CD8 T cells	1.609	1.343	1.510	1.207	0.976	1.098
NK cells	2.124	1.487	1.739	1.292	1.558	1.750
Monocytes	2.261	1.377	2.096	1.539	1.458	3.131

Mean values of fold change in HLA-E, HLA-ABC, and HLA-DR expression in different cell types of both MS patients (*n* = 14) and controls (*n* = 11) after 24 h of IFN-α and IFN-γ stimulation

**Fig. 2:**
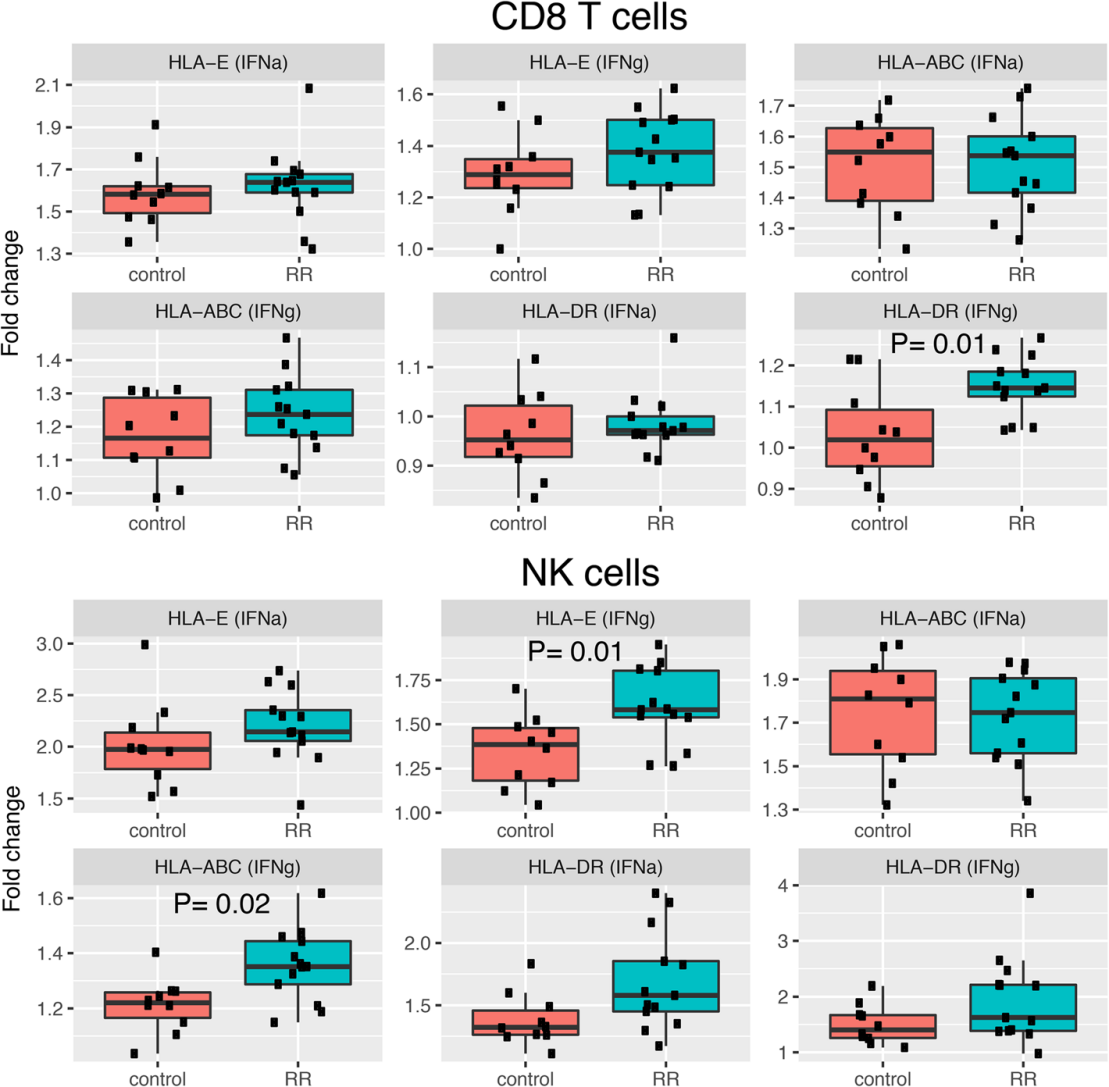
HLA expression is increased in MS patients upon interferon stimulation. PBMCs from RRMS patients and healthy controls were either left unstimulated or activated with IFN-α or IFN-γ for 24 h. Boxplots show the fold change in expression of HLA-E, HLA-ABC, and HLA-DR in CD8 T cells and NK cells in MS patients and controls. Each dot represents a sample. IFN-γ induced a higher HLA-DR expression in CD8 T cells and HLA-E and HLA-ABC expression in NK cells from MS patients compared to controls. Statistical differences were calculated using Wilcoxon test

**Fig. 3:**
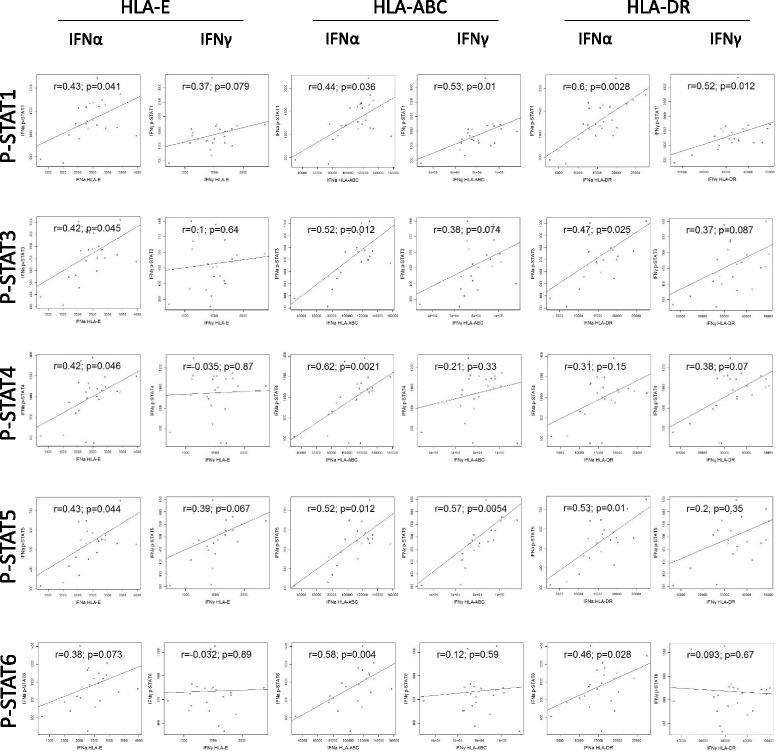
Interferon-induced HLA expression correlates with STAT protein phosphorylation in monocytes. Scatterplots show the correlation between mean fluorescence intensity (MFI) of each STAT protein and the expression levels of HLA-E, HLA-ABC, and HLA-DR after stimulation with IFN-α or INF-γ in monocytes from MS patients and controls. IFN-α induced HLA expression in monocytes correlates with levels of phosphorylated STAT proteins. Correlation between variables was calculated using Spearman’s rank correlation coefficient

In a subsequent analysis, we compared the increase in HLA expression between MS patients and healthy subjects in all cell subtypes. Notably, we only observed a higher fold change in HLA-DR expression in CD8 T cells and a higher fold change in HLA-ABC and HLA-E expression in NK cells after IFN-γ stimulation in MS patients compared to controls (Fig. 2). We also analyzed the correlation between HLA-E, HLA-ABC and HLA-DR expression and p-STAT proteins levels and we found that in B cells, p-STAT1 and p-STAT5 levels correlated with HLA expression after IFN-γ stimulation while p-STAT4 negatively correlated with HLA expression. Surprisingly, in NK cells, p-STAT3, p-STAT4 and p-STAT6 levels correlated negatively with HLA expression after IFN-γ stimulation (Additional file 10: Table S9). On the other hand, in monocytes, levels of phosphorylated STAT proteins were found consistently correlated with increased HLA expression after stimulation with both IFN-α and IFN-γ (Fig. 3).

### Antigen-specific response in EAE is mediated by p38MAPK and multiple STAT proteins

**Fig. 4:**
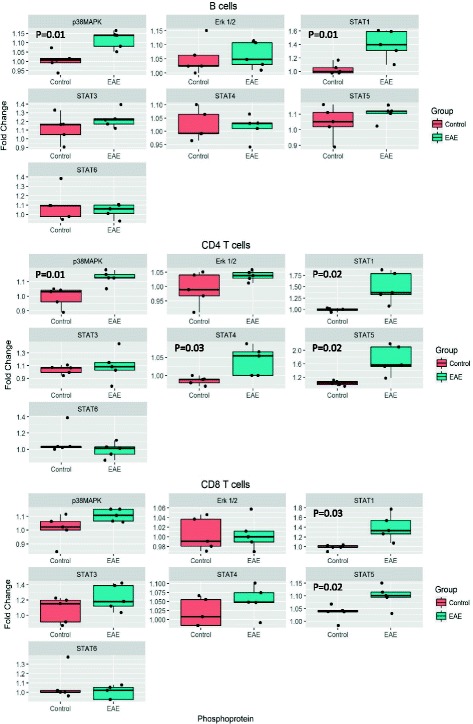
Antigen-specific response in EAE is mediated by STAT protein phosphorylation. Splenocytes from EAE and control mice were stimulated with MOG_35–55_ peptide for 24 h and levels of p-p38MAPK, p-Erk1/2, p-STAT1, p-STAT3, p-STAT4, p-STAT5, and p-STAT6 were evaluated. Boxplots show the fold change in protein phosphorylation compared to unstimulated cells. MOG_35–55_ stimulation induced phosphorylation of STAT1 in all cell types, p38MAPK in B cells and CD4 T cells, STAT5 in CD4 and CD8 T cells, and STAT4 in CD4 T cells of EAE mice but not control mice. Statistical differences were calculated using an unpaired *t* test

The results described above showing an increased response to activation in MS patients were generated by challenging PBMCs with non-specific stimuli. To confirm the observed signaling dysregulation in the context of neuroantigen-specific responses, we analyzed the levels of selected proteins in splenocytes isolated from MOG_35–55_-induced EAE mice and re-stimulated in vitro with the same peptide. An increase in phosphorylation of p38MAPK was observed in B cells (*p* = 0.016) and CD4 T cells (*p* = 0.008) of EAE mice as compared to mock injected animals (Fig. 4), along with an increase in p-STAT1 in all cell types following the same pattern observed in human PBMCs. In addition, pSTAT4 was also increased after MOG stimulation in CD4 T cells of EAE mice compared to controls (*p* = 0.02), consistently mimicking the response observed in MS patients. We also found an increase in STAT5 phosphorylation in CD4 T cells (*p* = 0.008). These results confirm the singularity of each cell compartment in responding to activation and show that some of the pathways that are aberrantly activated in the immune cells of MS patients are also activated in an antigen-specific manner in the EAE model.

## Discussion

In the present study, we developed and optimized a phosphoflow cytometry protocol to simultaneously assess the activation status of multiple signaling pathways in cryopreserved lymphocytes isolated from well-characterized, untreated MS patients. The analysis at baseline conditions revealed a general downregulation of phosphorylated signaling proteins in MS across all peripheral cellular components, in particular p38MAPK, with the exception of Akt and Cbl, which were found upregulated in MS patients. Since increased p38MAPK phosphorylation has been described in the CNS upon EAE induction [25], and its pharmacological blockade has shown to inhibit the differentiation of CD4 T cells into Th17 cells, both in MS and the animal model [26, 27], our results appear counterintuitive. However, the increase in p38MAPK and other phosphoproteins phosphorylation after in vitro stimulation was significantly higher in MS patients compared to controls, suggesting that immune cells from MS patients exhibit a higher propensity to activation, independent of the baseline levels of phosphorylated protein. Additionally, in response to IFN-α stimulation we observed an extensive upregulation in the levels of phosphorylated STAT proteins in MS patients compared to controls across all cell subsets but especially within the NK cell population, which has been implicated in MS pathogenesis [28, 29]. GWAS have identified SNPs in the proximity of *STAT* genes associated with increased risk of MS [3, 4, 21] and genetic depletion of these genes modulates EAE development [30–32]. Our study confirms the role of STAT proteins, in particular of STAT1, in immune cell activation of both human cells and splenocytes from EAE mice in response to the MOG immunogenic peptide.

Given that HLA expression can be modulated by interferons [22, 23, 33], we were also interested in investigating the association between increased STAT protein phosphorylation and HLA expression in response to interferon in MS patients and controls. Our results show a clear correlation between levels of STAT phosphorylation in monocytes, which are the primary antigen presenting cells in the blood, and the levels of HLA expression after stimulation with interferon but not at baseline conditions. Interestingly, no consistent correlations were found between levels of phosphorylated STAT proteins and HLA expression in B cells, which are also known to participate in antigen presentation, suggesting different regulation machineries for HLA expression in response to interferon across cell types. The results also indicate that monocytes are more susceptible to activation by interferon in MS patients. This exaggerated activation, reflected as an increased phosphorylation of STAT proteins, would lead to an upregulation of HLA expression in this cell type and, as a potential consequence, a dysregulation of the immune responses.

Two additional (negative) observations are worth discussing. A recent study proposed that genetic risk variants affect the activation of transcriptional factors [5]. The increased phosphorylation response that we observe in MS patients appears not to be related to the cumulative disease susceptibility variants each individual carries either globally (MSGB), stratified by relevant ontological pathways (MSPBphos, MSPBregphos), or the individual risk variants in the proximity of STAT genes. One limitation of our analysis, however, was the limited power of the dataset and consequent impaired capacity of detecting allelic variants effects on the endophenotype. Although the role of genetic variants in immune cell activation cannot be completely discarded, other mechanisms could explain the differential response observed in MS patients. Previous studies have shown that the levels of expression of IFN receptors (IFNAR1 and IFNAR2) correlate with the activation of some STAT proteins [34, 35]. We show that both IFNα and IFNγ can regulate HLA expression, but only IFNα-induced HLA expression correlates with STAT proteins phosphorylation. This could make us think that, indeed, a differential expression of IFNAR1 and IFNAR2 in the immune cells of MS patients could be driving the increased activation response observed in MS. However, this would not explain the increased phosphorylation observed in other proteins in response to H_2_O_2_ or PMA. This seems to indicate that changes in the levels or function of other kinases and phosphatases involved in regulation of protein phosphorylation might be playing a role in how molecules from multiple signaling cascades are responding to stimulation.

In addition, we did not find any differences on the protein phosphorylation profile between RRMS and SPMS patients. Even though the number of samples from SPMS patients and the corresponding RRMS patients was limited, these results are in agreement with the observed lack of association between levels of phosphorylated protein and EDSS. This result suggests that the phosphorylation response in immune cells is not associated with the severity of the disease and thus it does not change during disease progression.

Finally, EAE has been widely used to simulate multiple aspects of the disease. One of the main advantages of this model resides in the a priori knowledge of the antigen driving the autoimmune response. For that reason, we were interested in analyzing the phosphorylation profile of splenocytes from EAE mice against the MOG peptide, which could reveal the pathways activated during antigen-specific responses. The increased p-p38MAPK and p-STAT levels after MOG_35–55_ stimulation found in EAE mice mimics the response observed in the different human PBMCs cell compartments and supports the role of STAT proteins phosphorylation as previously proposed [36].

## Conclusions

Our results show that immune cells from MS patients are highly sensitive to different in vitro stimuli, especially to IFN-α through STAT proteins signaling. This dysregulation is observed in both relapsing and progressive clinical courses, and does not seem to be related to the genetic variants associated with the disease. Our findings also suggest that activation of the STAT pathway in immune cells can modify the expression of HLA in monocytes, which are the main antigen presenting cell in the blood, and this could lead to changes in the regulation of the immune response. The fact that each of the protein presents a different phosphorylation pattern in each individual cell type, suggests that the activation responses are selectively regulated. Therefore, further work to understand the effect of the activation of individual pathways will need to be focused on highly homogenous cell lineages or even single cells.

## Additional files

Additional file 1: **Figure S1.** Gating strategy to determine levels of phosphoprotein on different blood cell subsets. First, based on the combination of FSC and SSC, lymphocytes and monocytes were selected. Monocytes were identified as CD14+. T cells were identified by CD3+ staining then they were transferred to a new dot plot and were analyzed by CD4 staining. CD4 T cells were identified as CD3+ and CD4+ cells, while CD8 T cells as CD3+ and CD4− cells. B cells were identified as CD3− CD19+. The CD3− CD19− population was transferred to a new dot plot, and CD16low/− CD56+ cells were identified as NK cells. Table lists the antibodies used for human and mouse phosphoflow cytometry and HLA expression studies. (PDF 220 kb)

Additional file 2: **Table S1.** Comparison of levels of phosphorylated proteins between MS patients and controls in baseline conditions. Levels of phosphorylated proteins in each cell type in healthy controls and RRMS patients. Values represent the mean fluorescence intensity and standard deviation for each group. (DOCX 14 kb)

Additional file 3: **Table S2.** Comparison of fold change of protein phosphorylation between MS patients and controls after in vitro stimulation. Fold change in the levels phosphorylated proteins induced by in vitro stimulation in each cell type in healthy controls and RRMS patients. Values represent the mean fold change of phosphorylation levels and standard deviation for each group. (DOCX 15 kb)

Additional file 4: **Table S3.** Comparison of levels of phosphorylated proteins between MS patients and controls after in vitro stimulation. Levels of phosphorylated proteins in each cell type in healthy controls and RRMS patients. Values represent the mean fluorescence intensity and standard deviation for each group. (DOCX 14 kb)

Additional file 5: **Table S4.** Comparison of levels of p38MAPK, Erk1/2, STAT1, and STAT6 between MS patients and controls. Levels of selected proteins in each cell type in healthy controls and RRMS patients. Values represent the mean fluorescence intensity and standard deviation for each group. (DOCX 13 kb)

Additional file 6: **Table S5.** Comparison of levels of phosphorylated proteins between RRMS and SPMS patients in baseline conditions. Levels of phosphorylated proteins in each cell type in RRMS and SPMS patients. Values represent the mean fluorescence intensity and standard deviation for each group. (DOCX 14 kb)

Additional file 7: **Table S6.** Comparison of levels of phosphorylated proteins between RRMS and SPMS patients after in vitro stimulation. Levels of phosphorylated proteins in each cell type in RRMS and SPMS patients. Values represent the mean fluorescence intensity and standard deviation for each group. (DOCX 15 kb)

Additional file 8: **Table S7.** Correlation between MS genetic burden and MS risk loci with levels of phosphorylated proteins in different cell types (baseline). Correlation between baseline levels of phosphorylated proteins and the MSGB (MS genetic burden), MSPBphos (pathway burden of protein phosphorylation ontological family), or MSPBregphos (pathway burden of regulation of protein phosphorylation ontological family) in each cell type. Cor: Spearman coefficient; p: *p* values. Significant correlations are highlighted in bold. (DOCX 21 kb)

Additional file 9: **Table S8.** Correlation between MS genetic burden and MS risk loci with levels of phosphorylated proteins after in vitro stimulation in different cell types. Correlation between levels of phosphorylated proteins after in vitro stimulation and the MSGB (MS genetic burden), MSPBphos (pathway burden of protein phosphorylation ontological family), or MSPBregphos (pathway burden of regulation of protein phosphorylation ontological family) in each cell type analyzed. Cor: Spearman coefficient; p: *p* values. Significant correlations are highlighted in bold. (DOCX 17 kb)

Additional file 10: **Table S9.** Correlation between STAT phosphorylation and HLA-E, HLA-ABC, and HLA-DR expression in different cell populations. Correlation between levels of p-STAT1, p-STAT3, p-STAT4, p-STAT5, and p-STAT6 proteins and HLA-E, HLA-ABC, and HLA-DR expression after IFN-α or IFN-γ stimulation. Cor: Spearman coefficient; p: *p* values. Significant correlations are highlighted in bold. (DOCX 15 kb)

## Abbreviations

IFN-α: Interferon alpha
IFN-γ: Interferon gamma
JAK: Janus kinase
MFI: Mean fluorescence intensity
p38MAPK: p38 mitogen-activated protein kinase
PBMC: Peripheral blood mononuclear cells
PMA: Phorbol 12-myristate 13-acetate
STAT: Signal transducer and activator of transcription
