# Supplementary material for: Aberrant STAT phosphorylation signaling in peripheral blood mononuclear cells from multiple sclerosis patients
Source: J Neuroinflammation. 2018 Mar 7;15:72. doi: 10.1186/s12974-018-1105-9 (PMC5840794; doi:10.1186/s12974-018-1105-9)
Supplement: Supplementary file 1 — Figure S1. Gating strategy to determine levels of phosphoprotein on different blood cell subsets. First, based on the combination of FSC and SSC, lymphocytes and monocytes were selected. Monocytes were identified as CD14+. T cells were identified by CD3+ staining then they were transferred to a new dot plot and were analyzed by CD4 staining. CD4 T cells were identified as CD3+ and CD4+ cells, while CD8 T cells as CD3+ and CD4− cells. B cells were identified as CD3− CD19+. The CD3− CD19− population was transferred to a new dot plot, and CD16low/− CD56+ cells were identified as NK cells. Table lists the antibodies used for human and mouse phosphoflow cytometry and HLA expression studies. (PDF 220 kb) [file 12974_2018_1105_MOESM1_ESM.pdf]

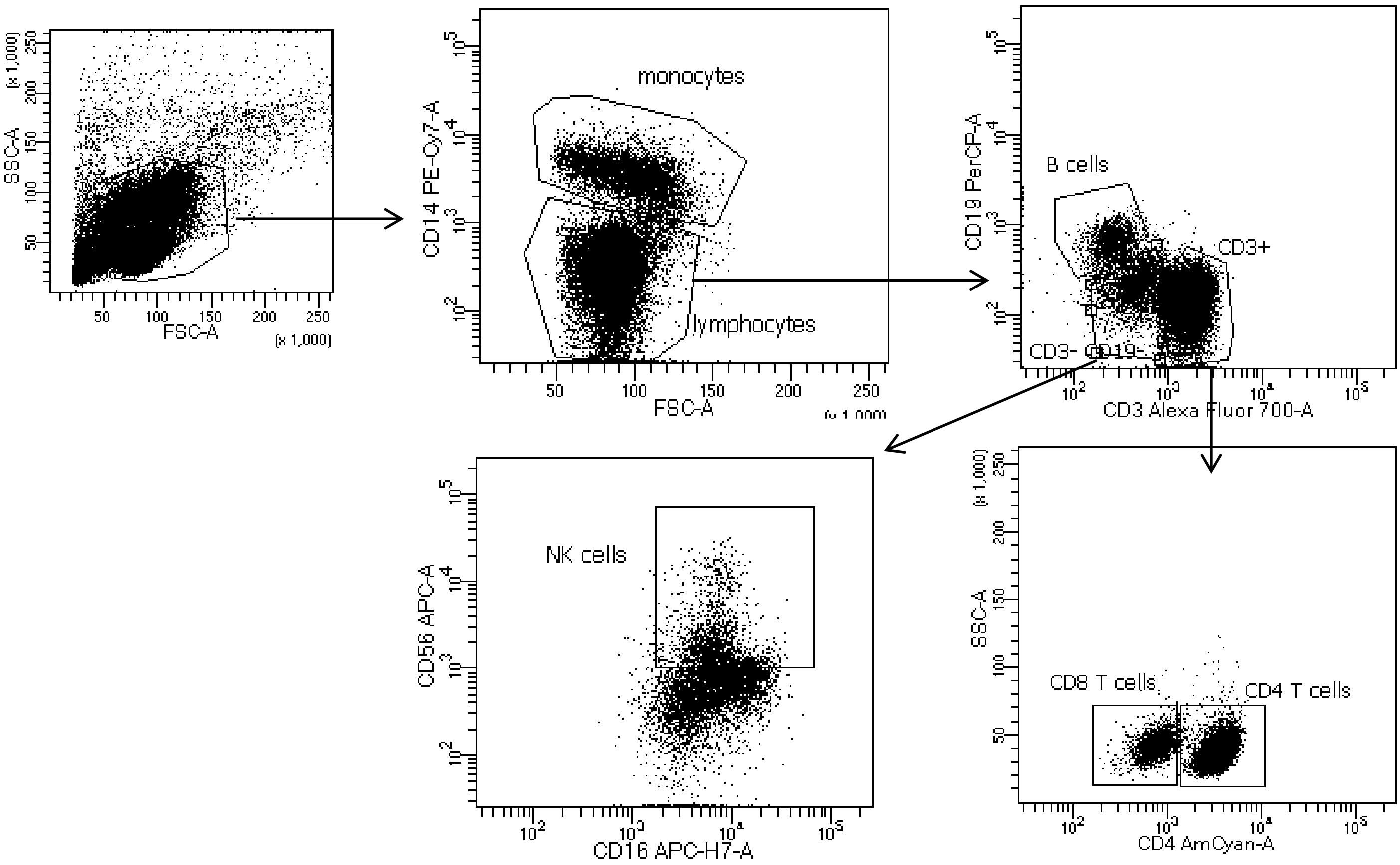

Molecule	Clone
**Human-CD3**	UCHT1 (BD biosciences)
**Human-CD4**	RPA-T4 (BD biosciences)
**Human-CD19**	HIB19 (BD biosciences)
**Human-CD14**	M5E2 (BD biosciences)
**Human-CD16**	B73.1 (BD biosciences)
**Human-CD56**	B159 (BD biosciences)
**Akt (pS473)**	M89-61 (BD biosciences)
**Btk (pY551)/ Itk (pY511)**	24a/BTK (BD biosciences)
**PLCγ1 (pY783)**	27/PLC (BD biosciences)
**c-Cbl (pY700)**	47/c-Cbl (BD biosciences)
**p38MAPK (pT180/pY182)**	36/p38 (BD biosciences)
**p38MAPK**	D13E1 (cell signaling)
**Erk1/2 (pT202/2Y204)**	20A (BD biosciences)
**Erk1/2**	137F5 (cell signaling)

Molecule	Clone
**Stat1 (pY701)**	14/P-STAT1 (BD biosciences)
**Stat3 (pY705)**	4/P-STAT3 (BD biosciences)
**Stat4 (pY693)**	28/P-STAT4 (BD biosciences)
**Stat5 (pY694)**	47/STAT5 (BD biosciences)
**Stat6 (pY641)**	18/P-STAT6 (BD biosciences)
**Stat1**	1/Stat1 (BD biosciences)
**Stat6**	23/Stat6 (BD biosciences)
**Anti-rabbit Alexa Fluor 488**	#4412 (cell signaling)
**HLA-E**	3D12 (Biolegend)
**HLA-ABC**	W6/32 (Biolegend)
**HLA-DR**	L243 (Biolegend)
**Mouse-CD3e**	145-2C11 (BD Pharmingen)
**Mouse-CD19**	ID3 (BD Pharmingen)
**Mouse-CD4**	GK1.5 (BD Pharmingen)
**Mouse-CD8a**	53-6.7 (BD Pharmingen)
