# Supplementary material for: Aberrant STAT phosphorylation signaling in peripheral blood mononuclear cells from multiple sclerosis patients
Source: J Neuroinflammation. 2018 Mar 7;15:72. doi: 10.1186/s12974-018-1105-9 (PMC5840794; doi:10.1186/s12974-018-1105-9)
Supplement: Supplementary file 2 — Table S1. Comparison of levels of phosphorylated proteins between MS patients and controls in baseline conditions. Levels of phosphorylated proteins in each cell type in healthy controls and RRMS patients. Values represent the mean fluorescence intensity and standard deviation for each group. (DOCX 14 kb) [file 12974_2018_1105_MOESM2_ESM.docx]

Table S1. Comparison of levels of phosphorylated proteins between MS patients and controls in baseline conditions

| Cell type | Group | Akt | Btk | Cbl | Erk1/2 | P38MAPK | PLCγ | STAT1 | STAT3 | STAT4 | STAT5 | STAT6 |
| --- | --- | --- | --- | --- | --- | --- | --- | --- | --- | --- | --- | --- |
| B cells | Control | 116.45 (19.30) | 117.04 (19.89) | 26.41 (5.83) | 108.99 (21.85) | 88.09 (20.41) | 43.35 (6.76) | 91.96 (17.74) | 72.86 (13.56) | 109.75 (27.05) | 112.39 (16.64) | 49.07 (8.34) |
|  | RR | 122.04 (14.35) | 103.65 (14.86) | 26.51 (4.23) | 103.65 (17.14) | 70.23 (7.38) | 40.02 (5.29) | 86.34 (9.10) | 69.26 (11.07) | 102.40 (20.49) | 104.57 (16.26) | 51.59 (14.38) |
|  | p-value | 0.236 | **0.006** | 0.910 | 0.201 | **5.16x10^-5^** | **0.041** | 0.164 | 0.277 | 0.271 | 0.083 | 0.782 |
| CD4 T cells | Control | 108.59 (24.42) | 101.94 (14.55) | 23.95 (4.75) | 86.84 (12.59) | 53.69 (9.01) | 37.75 (5.15) | 82.10 (10.35) | 59.39 (7.86) | 121.03 (34.83) | 107.09 (11.14) | 31.27 (4.29) |
|  | RR | 114.59 (12.09) | 100.96 (15.81) | 24.90 (4.63) | 87.66 (12.79) | 49.29 (4.89) | 36.57 (5.96) | 76.38 (8.08) | 57.49 (6.49) | 104.75 (17.78) | 105.99 (16.43) | 29.87 (4.30) |
|  | p-value | 0.495 | 0.682 | 0.396 | 0.853 | **0.010** | 0.515 | **0.014** | 0.366 | 0.120 | 0.526 | 0.217 |
| CD8 T cells | Control | 120.93 (17.00) | 105.61 (15.21) | 27.65 (5.22) | 105.18 (19.94) | 73.58 (15.18) | 47.12 (7.19) | 86.24 (11.45) | 67.55 (11.42) | 132.33 (33.07) | 116.49 (15.09) | 40.56 (5.35) |
|  | RR | 132.61 (11.41) | 100.04 (11.23) | 28.52 (4.41) | 104.28 (15.65) | 65.96 (6.76) | 44.20 (4.68) | 82.19 (7.70) | 65.47 (7.69) | 125.61 (21.53) | 108.44 (13.73) | 37.51 (3.40) |
|  | p-value | **0.003** | 0.068 | 0.408 | 0.922 | **0.022** | 0.182 | 0.112 | 0.292 | 0.328 | **0.039** | **0.018** |
| NK cells | Control | 141.07 (25.82) | 116.34 (17.83) | 27.70 (5.95) | 120.86 (18.13) | 100.35 (28.58) | 48.73 (4.81) | 103.02 (13.02) | 69.93 (11.80) | 156.87 (34.58) | 130.29 (17.32) | 47.56 (6.81) |
|  | RR | 159.45 (15.91) | 112.08 (13.72) | 27.66 (5.03) | 123.14 (16.75) | 73.48 (7.71) | 47.98 (5.59) | 93.44 (9.47) | 72.17 (9.75) | 150.04 (26.87) | 125.76 (16.91) | 43.89 (4.55) |
|  | p-value | **0.001** | 0.423 | 0.942 | 0.559 | **1.05x10^-5^** | 0.628 | **0.002** | 0.514 | 0.338 | 0.368 | **0.048** |
| Monocytes | Control | 334.77 (88.36) | 265.89 (44.14) | 69.67 (15.01) | 223.17 (28.74) | 448.77 (109.17) | 117.72 (18.06) | 209.59 (29.29) | 276.77 (59.96) | 371.48 (68.81) | 281.15 (46.16) | 333.82 (75.09) |
|  | RR | 307.25 (53.07) | 273.95 (42.93) | 72.75 (13.98) | 225.40 (27.18) | 376.96 (74.33) | 106.56 (9.94) | 184.10 (15.41) | 236.96 (36.73) | 415.10 (85.35) | 280.27 (39.24) | 331.52 (58.31) |
|  | p-value | 0.168 | 0.389 | 0.351 | 0.943 | **0.003** | **0.012** | **2.05x10^-4^** | **0.003** | **0.031** | 0.933 | 0.986 |

Levels of phosphorylated proteins in each cell type in healthy controls and RRMS patients. Values represent the mean fluorescence intensity and standard deviation for each group.
