# Supplementary material for: Aberrant STAT phosphorylation signaling in peripheral blood mononuclear cells from multiple sclerosis patients
Source: J Neuroinflammation. 2018 Mar 7;15:72. doi: 10.1186/s12974-018-1105-9 (PMC5840794; doi:10.1186/s12974-018-1105-9)
Supplement: Supplementary file 3 — Table S2. Comparison of fold change of protein phosphorylation between MS patients and controls after in vitro stimulation. Fold change in the levels phosphorylated proteins induced by in vitro stimulation in each cell type in healthy controls and RRMS patients. Values represent the mean fold change of phosphorylation levels and standard deviation for each group. (DOCX 15 kb) [file 12974_2018_1105_MOESM3_ESM.docx]

Table S2. Comparison of fold-change of protein phosphorylation between MS patients and controls after *in vitro* stimulation

| Cell type | Group | Akt | Btk | Cbl | Erk1/2 | P38MAPK | PLCγ | STAT1 | STAT3 | STAT4 | STAT5 | STAT6 |
| --- | --- | --- | --- | --- | --- | --- | --- | --- | --- | --- | --- | --- |
| B cells | Control | 2.68 (0.46) | 52.52 (15.88) | 35.56 (16.59) | 2.42 (0.43) | 3.34 (0.62) | 3.65 (0.65) | 4.51 (1.68) | 2.01 (0.60) | 1.15 (0.13) | 2.02 (0.58) | 3.09 (1.13) |
|  | RR | 2.82 (0.39) | 64.97 (11.91) | 42.18 (8.62) | 2.58 (0.39) | 4.18 (0.61) | 4.27 (0.67) | 5.85 (1.06) | 2.32 (0.36) | 1.18 (0.11) | 2.50 (0.52) | 3.43 (0.91) |
|  | p-value | 0.245 | **0.001** | 0.211 | 0.088 | **1.80x10^-6^** | **0.001** | **0.002** | **0.006** | 0.332 | **0.001** | 0.201 |
| CD4 T cells | Control | 2.67 (0.53) | 3.85 (1.07) | 10.96 (4.70) | 2.85 (0.54) | 5.04 (0.91) | 5.13 (1.08) | 9.76 (4.14) | 2.39 (0.66) | 2.62 (0.51) | 3.96 (1.40) | 4.74 (1.28) |
|  | RR | 2.65 (0.33) | 4.25 (0.75) | 10.09 (3.64) | 2.90 (0.47) | 5.69 (0.70) | 5.60 (1.01) | 13.25 (2.62) | 2.53 (0.47) | 3.12 (0.50) | 4.68 (1.05) | 5.85 (1.23) |
|  | p-value | 0.309 | 0.067 | 0.390 | 0.551 | **0.002** | 0.128 | **2.39x10^-4^** | 0.215 | **2.29x10^-5^** | **0.030** | **0.001** |
| CD8 T cells | Control | 2.36 (0.41) | 4.10 (1.07) | 7.77 (3.29) | 2.25 (0.36) | 3.63 (0.66) | 4.36 (0.75) | 6.82 (2.69) | 2.23 (0.57) | 2.93 (0.55) | 2.67 (0.80) | 4.27 (0.98) |
|  | RR | 2.16 (0.16) | 4.08 (0.62) | 6.64 (1.64) | 2.40 (0.42) | 3.75 (0.65) | 4.69 (0.72) | 10.00 (2.66) | 2.41 (0.45) | 3.60 (0.60) | 3.45 (1.03) | 5.63 (1.15) |
|  | p-value | **0.017** | 0.687 | 0.108 | 0.179 | 0.556 | 0.140 | **3.56x10^-5^** | 0.118 | **2.56x10^-6^** | **0.002** | **1.15x10^-5^** |
| NK cells | Control | 2.17 (0.41) | 6.21 (2.53) | 7.49 (3.31) | 2.15 (0.35) | 2.26 (0.55) | 2.78 (0.44) | 2.88 (1.11) | 1.79 (0.52) | 2.35 (0.71) | 1.30 (0.21) | 2.87 (0.72) |
|  | RR | 2.14 (0.32) | 7.13 (2.21) | 7.17 (1.98) | 2.25 (0.33) | 2.66 (0.41) | 3.08 (0.31) | 4.63 (0.70) | 1.85 (0.30) | 3.26 (0.62) | 1.43 (0.18) | 4.28 (0.69) |
|  | p-value | 0.653 | 0.133 | 0.488 | 0.145 | **4.68x10^-4^** | **0.001** | **2.04x10^-10^** | 0.587 | **1.60x10^-7^** | **0.006** | **1.80x10^-9^** |
| Monocytes | Control | 1.27 (0.25) | 4.28 (1.49) | 4.39 (1.39) | 2.01 (0.32) | 2.15 (0.52) | 1.76 (0.12) | 5.10 (2.23) | 1.63 (0.45) | 1.11 (0.10) | 1.48 (0.30) | 1.33 (0.29) |
|  | RR | 1.35 (0.24) | 4.51 (2.00) | 5.05 (1.10) | 2.36 (0.49) | 2.65 (0.52) | 1.93 (0.16) | 7.50 (1.36) | 2.38 (0.56) | 1.09 (0.08) | 1.77 (0.28) | 1.44 (0.24) |
|  | p-value | 0.151 | 0.913 | **0.007** | **0.003** | **1.22x10^-4^** | **1.04x10^-6^** | **2.15x10^-6^** | **3.89x10^-8^** | 0.324 | **1.38x10^-4^** | **0.049** |

Fold change in the levels phosphorylated proteins induced by *in vitro* stimulation in each cell type in healthy controls and RRMS patients. Values represent the mean fold change of phosphorylation levels and standard deviation for each group.
