# Supplementary material for: Aberrant STAT phosphorylation signaling in peripheral blood mononuclear cells from multiple sclerosis patients
Source: J Neuroinflammation. 2018 Mar 7;15:72. doi: 10.1186/s12974-018-1105-9 (PMC5840794; doi:10.1186/s12974-018-1105-9)
Supplement: Supplementary file 4 — Table S3. Comparison of levels of phosphorylated proteins between MS patients and controls after in vitro stimulation. Levels of phosphorylated proteins in each cell type in healthy controls and RRMS patients. Values represent the mean fluorescence intensity and standard deviation for each group. (DOCX 14 kb) [file 12974_2018_1105_MOESM4_ESM.docx]

Table S3. Comparison of levels of phosphorylated proteins between MS patients and controls after *in vitro* stimulation

| Cell type | Group | Akt | Btk | Cbl | Erk1/2 | P38MAPK | PLCγ | STAT1 | STAT3 | STAT4 | STAT5 | STAT6 |
| --- | --- | --- | --- | --- | --- | --- | --- | --- | --- | --- | --- | --- |
| B cells | Control | 313.42 (72.70) | 5987.83 (1537.98) | 945.34 (449.87) | 259.53 (42.50) | 285.64 (45.07) | 161.66 (34.10) | 404.13 (139.46) | 144.79 (39.95) | 126.52 (34.88) | 225.72 (59.90) | 153.82 (60.73) |
|  | RR | 338.07 (45.37) | 6637.44 (1070.26) | 1122.25 (256.57) | 265.73 (32.65) | 292.49 (41.60) | 165.87 (24.80) | 503.04 (81.23) | 157.94 (16.62) | 118.43 (25.07) | 258.80 (48.26) | 174.24 (55.27) |
|  | p-value | 0.251 | 0.153 | 0.209 | 0.677 | 0.589 | 0.753 | **0.001** | 0.094 | 0.293 | **0.012** | 0.279 |
| CD4 T cells | Control | 289.52 (78.37) | 398.57 (97.52) | 256.96 (104.01) | 246.75 (40.51) | 266.55 (47.64) | 193.82 (41.67) | 797.22 (332.26) | 141.84 (39.68) | 316.96 (82.04) | 414.37 (135.49) | 148.32 (41.14) |
|  | RR | 302.34 (30.13) | 421.10 (82.25) | 248.25 (102.61) | 252.66 (39.19) | 275.48 (38.04) | 200.16 (33.54) | 997.39 (157.23) | 145.75 (29.16) | 324.62 (55.60) | 487.55 (104.44) | 169.90 (40.51) |
|  | p-value | 0.746 | 0.449 | 0.808 | 0.542 | 0.566 | 0.631 | **0.009** | 0.560 | 0.972 | **0.024** | **0.038** |
| CD8 T cells | Control | 277.88 (62.59) | 431.84 (108.71) | 213.23 (89.48) | 230.90 (32.99) | 262.33 (53.58) | 203.80 (36.38) | 578.03 (218.48) | 151.71 (34.31) | 387.06 (89.51) | 305.37 (75.08) | 173.19 (41.31) |
|  | RR | 288.00 (29.65) | 411.38 (67.04) | 186.68 (54.02) | 247.45 (34.46) | 247.56 (41.34) | 206.24 (32.87) | 807.72 (200.41) | 154.44 (28.20) | 447.46 (70.24) | 372.92 (100.07) | 206.10 (47.66) |
|  | p-value | 0.726 | 0.232 | 0.219 | 0.056 | 0.282 | 0.897 | **6.47x10^-5^** | 1.000 | **0.007** | **0.004** | **0.004** |
| NK cells | Control | 303.90 (41.42) | 714.06 (311.09) | 207.06 (101.02) | 136.49 (26.91) | 257.61 (28.15) | 216.43 (43.14) | 292.29 (118.34) | 126.89 (38.66) | 376.80 (132.81) | 168.00 (25.40) | 135.83 (34.89) |
|  | RR | 342.18 (65.94) | 808.47 (280.05) | 194.65 (60.66) | 148.32 (20.48) | 274.61 (37.85) | 196.05 (39.06) | 429.95 (70.15) | 134.88 (24.58) | 477.68 (64.87) | 178.90 (25.70) | 188.04 (40.16) |
|  | p-value | **0.027** | 0.167 | 0.762 | 0.051 | **0.040** | 0.060 | **2.50x10^-6^** | 0.269 | **0.001** | 0.091 | **3.19x10^-6^** |
| Monocytes | Control | 407.61 (52.98) | 1141.02 (436.68) | 298.83 (88.15) | 446.97 (60.19) | 926.52 (138.12) | 205.57 (23.18) | 1031.06 (398.06) | 456.10 (138.82) | 417.11 (95.92) | 414.97 (101.99) | 434.34 (102.91) |
|  | RR | 412.26 (43.48) | 1237.04 (499.11) | 363.98 (79.89) | 532.43 (98.06) | 975.72 (149.48) | 205.26 (19.24) | 1373.56 (255.14) | 555.58 (115.17) | 451.01 (81.52) | 494.12 (95.84) | 482.33 (93.91) |
|  | p-value | 0.812 | 0.460 | **0.006** | **1.04x10^-4^** | 0.119 | 0.966 | **1.34x10^-4^** | **0.003** | 0.163 | **0.003** | **0.050** |

Levels of phosphorylated proteins in each cell type in healthy controls and RRMS patients. Values represent the mean fluorescence intensity and standard deviation for each group.
