# Supplementary material for: Aberrant STAT phosphorylation signaling in peripheral blood mononuclear cells from multiple sclerosis patients
Source: J Neuroinflammation. 2018 Mar 7;15:72. doi: 10.1186/s12974-018-1105-9 (PMC5840794; doi:10.1186/s12974-018-1105-9)
Supplement: Supplementary file 5 — Table S4. Comparison of levels of p38MAPK, Erk1/2, STAT1, and STAT6 between MS patients and controls. Levels of selected proteins in each cell type in healthy controls and RRMS patients. Values represent the mean fluorescence intensity and standard deviation for each group. (DOCX 13 kb) [file 12974_2018_1105_MOESM5_ESM.docx]

Table S4. Comparison of levels of p38MAPK, Erk1/2, STAT1 and STAT6 between MS patients and controls

| Cell type | Group | P38MAPK | Erk1/2 | STAT1 | STAT6 |
| --- | --- | --- | --- | --- | --- |
| B cells | Control | 3181.33 (1359.92) | 12910.29 (2976.91) | 382.81 (237.65) | 338.67 (118.69) |
|  | RR | 2932.90 (943.55) | 13154.57 (1984.50) | 355.05 (185.23) | 331.43 (78.43) |
|  | p-value | 0.372 | 0.920 | 0.501 | 0.734 |
| CD4 T cells | Control | 4093.62 (1552.61) | 13916.93 (3049.63) | 1199.24 (547.22) | 287.33 (102.83) |
|  | RR | 3903.33 (1220.33) | 13909.50 (1602.23) | 1060.95 (244.05) | 273.14 (57.16) |
|  | p-value | 0.584 | 0.425 | 0.941 | 0.706 |
| CD8 T cells | Control | 4543.09 (1774.21) | 14755.86 (3478.32) | 903.67 (543.10) | 267.76 (97.01) |
|  | RR | 4297.09 (1212.10) | 14704.69 (1860.13) | 752.76 (238.74) | 254.95 (51.71) |
|  | p-value | 0.618 | 0.359 | 0.842 | 0.792 |
| NK cells | Control | 7658.90 (3136.23) | 20221.57 (4581.84) | 675.14 (432.18) | 351.48 (127.27) |
|  | RR | 7391.38 (1961.48) | 19775.93 (2743.04) | 576.14 (174.73) | 335.28 (72.07) |
|  | p-value | 0.440 | 0.728 | 0.941 | 0.880 |
| Monocytes | Control | 15034.38 (5188.18) | 26887.33 (5831.05) | 962.33 (531.66) | 590.38 (200.52) |
|  | RR | 15240.38 (4250.39) | 27640.19 (3859.42) | 904.90 (224.35) | 625.48 (159.65) |
|  | p-value | 1.000 | 0.383 | 0.567 | 0.359 |

Levels of selected proteins in each cell type in healthy controls and RRMS patients. Values represent the mean fluorescence intensity and standard deviation for each group.
