# Supplementary material for: Aberrant STAT phosphorylation signaling in peripheral blood mononuclear cells from multiple sclerosis patients
Source: J Neuroinflammation. 2018 Mar 7;15:72. doi: 10.1186/s12974-018-1105-9 (PMC5840794; doi:10.1186/s12974-018-1105-9)
Supplement: Supplementary file 6 — Table S5. Comparison of levels of phosphorylated proteins between RRMS and SPMS patients in baseline conditions. Levels of phosphorylated proteins in each cell type in RRMS and SPMS patients. Values represent the mean fluorescence intensity and standard deviation for each group. (DOCX 14 kb) [file 12974_2018_1105_MOESM6_ESM.docx]

Table S5. Comparison of levels of phosphorylated proteins between RRMS and SPMS patients in baseline conditions

| Cell type | Group | Akt | Btk | Cbl | Erk1/2 | P38MAPK | PLCγ | STAT1 | STAT3 | STAT4 | STAT5 | STAT6 |
| --- | --- | --- | --- | --- | --- | --- | --- | --- | --- | --- | --- | --- |
| B cells | RRMS | 245.78 (57.05) | 144.44 (25.23) | 36.78 (4.41) | 178.78 (37.02) | 192.67 (54.16) | 87.22 (17.43) | 149.44 (41.14) | 174.22 (49.25) | 171.44 (31.39) | 200.00 (38.89) | 90.44 (19.49) |
|  | SPMS | 259.20 (81.84) | 145.60 (25.75) | 39.30 (6.65) | 173.90 (33.12) | 202.00 (67.80) | 92.00 (21.15) | 139.90 (38.57) | 169.20 (63.93) | 164.30 (38.78) | 187.50 (41.92) | 97.00 (42.85) |
|  | p-value | 0.744 | 0.414 | 0.434 | 0.595 | 0.870 | 0.682 | 0.567 | 0.775 | 0.683 | 0.624 | 0.967 |
| CD4 T cells | RRMS | 249.33 (69.72) | 131.44 (22.61) | 35.89 (4.99) | 172.89 (38.54) | 129.11 (34.18) | 78.67 (22.89) | 153.11 (44.43) | 154.44 (44.08) | 181.44 (47.89) | 223.56 (60.74) | 65.22 (10.35) |
|  | SPMS | 270.50 (79.23) | 142.60 (27.18) | 40.20 (4.39) | 175.60 (32.65) | 140.10 (39.60) | 92.00 (25.17) | 152.50 (37.42) | 161.60 (50.56) | 184.00 (47.09) | 226.20 (56.70) | 71.10 (8.86) |
|  | p-value | 0.568 | 0.220 | 0.053 | 0.902 | 0.414 | 0.153 | 0.870 | 0.653 | 0.902 | 0.967 | 0.236 |
| CD8 T cells | RRMS | 276.11 (63.40) | 129.56 (17.63) | 38.22 (3.49) | 188.11 (40.40) | 165.00 (37.39) | 95.67 (20.54) | 160.44 (41.83) | 153.78 (43.12) | 220.78 (55.08) | 220.00 (55.08) | 82.89 (9.06) |
|  | SPMS | 289.50 (77.12) | 138.80 (19.80) | 41.40 (5.91) | 186.50 (35.35) | 165.90 (43.02) | 108.80 (24.57) | 156.00 (37.82) | 161.90 (46.06) | 215.40 (50.06) | 221.20 (53.33) | 84.50 (9.56) |
|  | p-value | 0.683 | 0.165 | 0.283 | 0.806 | 0.935 | 0.120 | 0.775 | 0.653 | 0.653 | 0.838 | 0.806 |
| NK cells | RRMS | 326.89 (61.42) | 146.00 (14.95) | 41.78 (4.84) | 222.78 (41.09) | 213.56 (71.90) | 103.00 (18.98) | 185.33 (46.13) | 195.78 (65.18) | 265.56 (59.23) | 250.22 (50.01) | 96.67 (11.50) |
|  | SPMS | 348.40 (99.81) | 154.70 (32.18) | 42.30 (5.14) | 222.30 (29.12) | 213.30 (62.50) | 127.90 (55.25) | 179.80 (40.78) | 203.30 (63.91) | 263.00 (58.23) | 243.20 (45.70) | 95.00 (8.33) |
|  | p-value | 0.744 | 0.487 | 0.967 | 0.838 | 0.838 | 0.205 | 0.744 | 0.744 | 0.967 | 0.775 | 0.623 |
| Monocytes | RRMS | 717.78 (228.19) | 396.33 (88.12) | 122.56 (18.39) | 380.22 (71.16) | 1,074.78 (290.10) | 242.56 (38.62) | 320.44 (82.25) | 580.89 (170.53) | 718.78 (126.09) | 480.11 (107.88) | 623.89 (103.17) |
|  | SPMS | 813.20 (344.25) | 381.70 (73.38) | 114.80 (13.41) | 383.80 (80.01) | 1,232.60 (509.18) | 255.70 (56.86) | 310.20 (69.03) | 599.80 (220.65) | 720.00 (162.25) | 466.20 (119.16) | 545.10 (96.98) |
|  | p-value | 0.806 | 0.935 | 0.203 | 0.870 | 0.568 | 0.683 | 0.595 | 0.935 | 0.744 | 0.683 | 0.165 |

Levels of phosphorylated proteins in each cell type in RRMS and SPMS patients. Values represent the mean fluorescence intensity and standard deviation for each group.
