# Supplementary material for: Aberrant STAT phosphorylation signaling in peripheral blood mononuclear cells from multiple sclerosis patients
Source: J Neuroinflammation. 2018 Mar 7;15:72. doi: 10.1186/s12974-018-1105-9 (PMC5840794; doi:10.1186/s12974-018-1105-9)
Supplement: Supplementary file 7 — Table S6. Comparison of levels of phosphorylated proteins between RRMS and SPMS patients after in vitro stimulation. Levels of phosphorylated proteins in each cell type in RRMS and SPMS patients. Values represent the mean fluorescence intensity and standard deviation for each group. (DOCX 15 kb) [file 12974_2018_1105_MOESM7_ESM.docx]

Table S6. Comparison of levels of phosphorylated proteins between RRMS and SPMS patients after *in vitro* stimulation

| Cell type | Group | Akt | Btk | Cbl | Erk1/2 | P38MAPK | PLCγ | STAT1 | STAT3 | STAT4 | STAT5 | STAT6 |
| --- | --- | --- | --- | --- | --- | --- | --- | --- | --- | --- | --- | --- |
| B cells | RRMS | 613.89 (134.48) | 6822.00 (3121.36) | 1586.89 (885.13) | 410.33 (41.11) | 617.11 (201.22) | 309.89 (94.33) | 474.22 (118.53) | 382.44 (80.07) | 190.89 (34.73) | 318.78 (62.86) | 319.00 (91.03) |
|  | SPMS | 599.10 (205.77) | 7001.00 (2420.48) | 1658.00 (846.01) | 381.80 (78.60) | 576.40 (159.56) | 307.60 (110.82) | 472.70 (122.20) | 393.00 (138.47) | 190.40 (55.47) | 311.90 (91.68) | 338.80 (126.44) |
|  | p-value | 0.568 | 0.935 | 0.744 | 0.462 | 0.462 | 0.967 | 0.870 | 0.935 | 0.806 | 0.744 | 0.935 |
| CD4 T cells | RRMS | 559.67 (82.45) | 445.11 (125.40) | 351.11 (151.15) | 424.11 (43.31) | 618.22 (177.65) | 395.44 (97.81) | 857.22 (283.64) | 382.22 (76.11) | 666.56 (158.43) | 599.56 (120.22) | 319.89 (96.68) |
|  | SPMS | 559.40 (159.85) | 460.70 (150.51) | 391.40 (146.86) | 404.80 (73.67) | 637.00 (170.61) | 427.00 (98.02) | 901.00 (190.24) | 401.10 (105.44) | 742.80 (193.41) | 623.90 (172.16) | 360.00 (112.14) |
|  | p-value | 0.624 | 0.744 | 0.624 | 0.624 | 0.935 | 0.568 | 1.000 | 0.870 | 0.327 | 0.462 | 0.514 |
| CD8 T cells | RRMS | 550.22 (78.68) | 497.22 (138.88) | 319.56 (132.75) | 394.22 (44.72) | 539.67 (168.31) | 429.78 (85.55) | 654.33 (217.42) | 364.44 (73.93) | 898.00 (207.43) | 445.22 (70.19) | 382.33 (111.09) |
|  | SPMS | 548.00 (146.47) | 494.60 (140.47) | 346.40 (123.02) | 374.70 (72.59) | 551.70 (183.49) | 450.70 (96.26) | 709.60 (231.09) | 375.80 (111.96) | 929.10 (253.04) | 472.40 (145.16) | 401.10 (130.46) |
|  | p-value | 0.870 | 0.967 | 0.806 | 0.595 | 1.000 | 0.540 | 0.624 | 0.806 | 0.624 | 0.414 | 0.683 |
| NK cells | RRMS | 586.67 (165.18) | 813.44 (357.17) | 311.56 (177.60) | 486.56 (41.71) | 490.33 (135.01) | 303.22 (59.44) | 465.78 (121.04) | 352.67 (89.30) | 838.67 (216.04) | 292.78 (55.58) | 332.00 (97.53) |
|  | SPMS | 606.60 (175.63) | 850.60 (274.97) | 381.80 (155.65) | 453.10 (68.10) | 501.20 (74.17) | 307.50 (56.30) | 438.00 (90.03) | 376.20 (111.82) | 837.70 (170.90) | 286.60 (59.43) | 323.80 (81.52) |
|  | p-value | 0.744 | 0.870 | 0.221 | 0.165 | 0.806 | 0.653 | 0.595 | 0.744 | 0.935 | 0.870 | 0.870 |
| Monocytes | RRMS | 886.44 (109.82) | 1197.89 (426.64) | 437.44 (153.58) | 648.89 (109.49) | 2520.78 (430.26) | 470.11 (58.87) | 1219.78 (393.71) | 1213.78 (293.08) | 784.78 (129.42) | 671.56 (132.58) | 948.11 (199.56) |
|  | SPMS | 930.90 (183.46) | 1409.70 (449.76) | 513.20 (126.76) | 665.50 (137.82) | 2595.70 (614.83) | 487.00 (60.11) | 1231.90 (416.71) | 1257.30 (457.90) | 809.30 (211.38) | 672.10 (163.33) | 920.60 (213.25) |
|  | p-value | 0.806 | 0.462 | 0.462 | 0.683 | 0.870 | 0.653 | 0.806 | 0.806 | 0.514 | 0.744 | 0.870 |

Levels of phosphorylated proteins in each cell type in RRMS and SPMS patients. Values represent the mean fluorescence intensity and standard deviation for each group.
