# Supplementary material for: Aberrant STAT phosphorylation signaling in peripheral blood mononuclear cells from multiple sclerosis patients
Source: J Neuroinflammation. 2018 Mar 7;15:72. doi: 10.1186/s12974-018-1105-9 (PMC5840794; doi:10.1186/s12974-018-1105-9)
Supplement: Supplementary file 8 — Table S7. Correlation between MS genetic burden and MS risk loci with levels of phosphorylated proteins in different cell types (baseline). Correlation between baseline levels of phosphorylated proteins and the MSGB (MS genetic burden), MSPBphos (pathway burden of protein phosphorylation ontological family), or MSPBregphos (pathway burden of regulation of protein phosphorylation ontological family) in each cell type. Cor: Spearman coefficient; p: p values. Significant correlations are highlighted in bold. (DOCX 21 kb) [file 12974_2018_1105_MOESM8_ESM.docx]

Table S7. Correlation between MS genetic burden and MS risk loci with levels of phosphorylated proteins in different cell types (baseline)

| Cell type | MS risk | Akt | Btk | Cbl | Erk1/2 | P38MAPK | PLCγ | STAT1 | STAT3 | STAT4 | STAT5 | STAT6 |
| --- | --- | --- | --- | --- | --- | --- | --- | --- | --- | --- | --- | --- |
| B cells | MSGB | cor=−0.08; p=0.674 | cor=−0.36; p=0.056 | cor=−0.02; p=0.903 | cor=−0.13; p=0.484 | cor=0.026; p=0.889 | cor=−0.11; p=0.568 | cor=−0.06; p=0.754 | cor=−0.03; p=0.892 | cor=−0.15; p=0.421 | cor=−0.06; p=0.779 | cor=0.18; p=0.362 |
|  | Prot Phos | cor=0.04; p=0.829 | cor=−0.15; p=0.434 | cor=−0.16; p=0.397 | cor=0.05; p=0.797 | cor=0.16; p=0.374 | cor=−0.11; p=0.575 | cor=0.05; p=0.814 | cor=−0.05; p=0.795 | cor=−0.01; p=0.954 | cor=−0.12; p=0.557 | cor=0.35; p=0.071 |
|  | Prot reg phos | cor=0.07; p=0.705 | cor=−0.25; p=0.197 | cor=0.08; p=0.669 | cor=0.06; p=0.732 | cor=−0.008; p=0.967 | cor=0.15; p=0.448 | cor=0.09; p=0.669 | cor=0.14; p=0.483 | cor=−0.06; p=0.742 | cor=0.11; p=0.572 | cor=0.17; p=0.373 |
|  | STAT1_4 | - | - | - | - | - | - | cor=−0.13; p=0.947 | - | **cor=−0.39; p=0.038** | - | - |
|  | STAT3_5 | - | - | - | - | - | - | - | cor=−0.06; p=0.772 | - | cor=−0.09; p=0.662 | - |
|  | STAT6 | - | - | - | - | - | - | **-** | - | - | **-** | cor=0.02; p=0.912 |
| CD4 T cells | MSGB | cor=−0.16; p=0.385 | cor=−0.20; p=0.286 | cor=−0.33; p=0.986 | cor=−0.27; p=0.138 | cor=−0.028; p=0.884 | cor=−0.11; p=0.545 | cor=−0.33; p=0.085 | cor=−0.13; p=0.516 | cor=−0.07; p=0.720 | cor=−0.16; p=0.420 | cor=0.20; p=0.311 |
|  | Prot Phos | cor=−0.04; p=0.826 | cor=−0.16; p=0.400 | cor=−0.22; p=0.233 | cor=−0.18; p=0.322 | cor=−0.02; p=0.914 | cor=−0.09; p=0.627 | **cor=−0.46; p=0.012** | cor=−0.14; p=0.466 | cor=−0.27; p=0.892 | cor=−0.13; p=0.510 | cor=0.03; p=0.888 |
|  | Prot reg phos | cor=−0.04; p=0.838 | cor=−0.18; p=0.345 | cor=0.04; p=0.842 | cor=−0.11; p=0.541 | cor=−0.12; p=0.522 | cor=0.17; p=0.365 | **cor=−0.401; p=0.031** | cor=−0.27; p=0.168 | cor=0.04; p=0.845 | cor=0.21; p=0.266 | cor=0.23; p=0.248 |
|  | STAT1_4 | - | - | - | - | - | - | cor=0.266; p=0.162 | - | cor=−0.33; p=0.082 | - | - |
|  | STAT3_5 | - | - | - | - | - | - | - | cor=−0.15; p=0.460 | - | cor=−0.07; p=0.733 | - |
|  | STAT6 | - | - | - | - | - | - | **-** | - | - | **-** | cor=0.25; p=0.194 |
| CD8 T cells | MSGB | cor=−0.16; p=0.389 | cor=−0.22; p=0.260 | cor=0.09; p=0.629 | cor=−0.24; p=0.184 | cor=0.02; p=0.923 | cor=−0.12; p=0.516 | cor=−0.36; p=0.053 | cor=−0.27; p=0.151 | cor=−0.09; p=0.626 | cor=−0.10; p=0.619 | cor=0.06; p=0.761 |
|  | Prot Phos | cor=0.08; p=0.654 | cor=−0.20; p=0.305 | cor=−0.16; p=0.375 | cor=−0.04; p=0.815 | cor=0.13; p=0.477 | cor=−0.16; p=0.397 | cor=−0.35; p=0.065 | cor=−0.32; p=0.090 | cor=−0.02; p=0.938 | cor=−0.19; p=0.347e | cor=0.05; p=0.778 |
|  | Prot reg phos | cor=−0.001; p=0.994 | cor=−0.27; p=0.156 | cor=0.03; p=0.872 | cor=−0.004; p=0.981 | cor=−0.01; p=0.957 | cor=0.16; p=0.409 | **cor=−0.48; p=0.008** | **cor=−0.43; p=0.019** | cor=−0.39; p=0.836 | cor=0.22; p=0.270 | cor=0.19; p=0.343 |
|  | STAT1_4 | - | - | - | - | - | - | cor=0.23; p=0.227 | - | **cor=−0.40; p=0.030** | - | - |
|  | STAT3_5 | - | - | - | - | - | - | - | cor=−0.22; p=0.253 | - | cor=−0.17; p=0.406 | - |
|  | STAT6 | - | - | - | - | - | - | **-** | - | **-** | **-** | cor=0.22; p=0.256 |
| NK cells | MSGB | cor=−0.37; p=0.059 | cor=−0.37; p=0.065 | cor=0.17; p=0.391 | cor=−0.13; p=0.528 | cor=0.07; p=0.736 | cor=−0.17; p=0.383 | cor=−0.24; p=0.260 | cor=−0.12; p=0.584 | cor=−0.18; p=0.395 | cor=−0.05; p=0.815 | cor=0.15; p=0.496 |
|  | Prot Phos | cor=0.08; p=0.683 | cor=−0.30; p=0.140 | cor=−0.189; p=0.344 | cor=−0.007; p=0.974 | cor=0.04; p=0.834 | cor=−0.08; p=0.709 | cor=−0.38; p=0.066 | cor=−0.20; p=0.348 | cor=−0.05; p=0.827 | cor=−0.19; p=0.385 | cor=0.02; p=0.924 |
|  | Prot reg phos | cor=−0.06; p=0.775 | cor=−0.25; p=0.226 | cor=0.32; p=0.104 | cor=0.09; p=0.655 | cor=0.04; p=0.855 | cor=0.23; p=0.245 | cor=−0.28; p=0.187 | cor=0.09; p=0.671 | cor=0.10; p=0.630 | cor=0.23; p=0.287 | **cor=0.53; p=0.008** |
|  | STAT1_4 | - | - | - | - | - | - | cor=0.08; p=0.711 | - | cor=−0.34; p=0.104 | - | - |
|  | STAT3_5 | - | - | - | - | - | - | - | cor=−0.39; p=0.061 | - | cor=−0.33; p=0.113 | - |
|  | STAT6 | **-** | - | - | - | **-** | - | **-** | - | **-** | - | **cor=0.67; p=3.06e−04** |
| Monocytes | MSGB | cor=−0.06; p=0.729 | cor=−0.16; p=0.389 | cor=0.003; p=0.986 | cor=−0.21; p=0.268 | cor=0.08; p=0.657 | cor=−0.10; p=0.580 | cor=−0.32; p=0.097 | cor=−0.25; p=0.188 | cor=−0.09; p=0.626 | cor=−0.08; p=0.681 | cor=0.18; p=0.349 |
|  | MSPBphos | cor=0.21; p=0.261 | cor=−0.02; p=0.913 | cor=0.09; p=0.612 | cor=−0.007; p=0.970 | cor=0.20; p=2.82e−01 | cor=−0.08; p=0.651 | cor=−0.17; p=0.392 | cor=−0.21; p=0.276 | cor=−0.08; p=0.695 | cor=−0.21; p=0.274 | cor=0.16; p=0.403 |
|  | MSPBregphos | cor=−0.17; p=0.359 | cor=0.03; p=0.861 | cor=0.09; p=0.613 | cor=−0.03; p=0.888 | cor=−0.15; p=0.404 | cor=0.17; p=0.371 | cor=−0.18; p=0.359 | cor=−0.30; p=0.110 | cor=−0.08; p=0.682 | cor=0.18; p=0.354 | cor=−0.01; p=0.954 |
|  | STAT1_4 | - | - | - | - | - | - | cor=0.01; p=0.952 | - | cor=−0.26; p=0.181 | - | - |
|  | STAT3_5 | - | - | - | - | - | - | - | cor=0.14; p=0.462 | - | cor=−0.22; p=0.253 | - |
|  | STAT6 | - | - | **-** | - | - | - | **-** | **-** | - | **-** | cor=0.14; p=0.476 |

Correlation between baseline levels of phosphorylated proteins and the MSGB (MS genetic burden), MSPBphos (pathway burden of protein phosphorylation ontological family) or MSPBregphos (pathway burden of regulation of protein phosphorylation ontological family) in each cell type. Cor: Spearman coefficient; p: p-values. Significant correlations are highlighted in bold.
