# Supplementary material for: Aberrant STAT phosphorylation signaling in peripheral blood mononuclear cells from multiple sclerosis patients
Source: J Neuroinflammation. 2018 Mar 7;15:72. doi: 10.1186/s12974-018-1105-9 (PMC5840794; doi:10.1186/s12974-018-1105-9)
Supplement: Supplementary file 9 — Table S8. Correlation between MS genetic burden and MS risk loci with levels of phosphorylated proteins after in vitro stimulation in different cell types. Correlation between levels of phosphorylated proteins after in vitro stimulation and the MSGB (MS genetic burden), MSPBphos (pathway burden of protein phosphorylation ontological family), or MSPBregphos (pathway burden of regulation of protein phosphorylation ontological family) in each cell type analyzed. Cor: Spearman coefficient; p: p values. Significant correlations are highlighted in bold. (DOCX 17 kb) [file 12974_2018_1105_MOESM9_ESM.docx]

Table S8. Correlation between MS genetic burden and MS risk loci with levels of phosphorylated proteins after *in vitro* stimulation in different cell types

| Cell type | MS risk | Akt | Btk | Cbl | Erk1/2 | P38MAPK | PLCγ | STAT1 | STAT3 | STAT4 | STAT5 | STAT6 |
| --- | --- | --- | --- | --- | --- | --- | --- | --- | --- | --- | --- | --- |
| B cells | MSGB | cor=0.08; p=0.660 | cor=−0.27; p=0.150 | cor=−0.23; p=0.214 | cor=−0.19; p=0.340 | cor=−0.15; p=0.428 | cor=0.32; p=0.087 | cor=−0.25; p=0.208 | cor=−0.008; p=0.970 | cor=−0.12; p=0.547 | cor=−0.10; p=0.603 | cor=0.006; p=0.977 |
|  | Prot Phos | cor=−0.02; p=0.929 | cor=−0.27; p=0.151 | cor=−0.15; p=0.442 | cor=−0.25; p=0.191 | cor=−0.24; p=0.194 | cor=0.04; p=0.815 | cor=−0.13; p=0.539 | cor=−0.31; p=0.129 | cor=−0.08; p=0.674 | cor=0.05; p=0.813 | cor=0.06; p=0.741 |
|  | Prot reg phos | cor=0.003; p=0.985 | cor=−0.19; p=0.313 | cor=−0.82; p=0.667 | cor=−0.15; p=0.438 | cor=−0.10; p=0.610 | cor=0.17; p=0.364 | cor=0.10; p=0.610 | cor=0.13; p=0.514 | cor=0.02; p=0.898 | cor=0.21; p=0.290 | cor=0.18; p=0.346 |
|  | STAT1_4 | - | - | - | - | - | - | cor=0.15; p=0.474 | - | cor=−0.21; p=0.274 | - | - |
|  | STAT3_5 | - | - | - | - | - | - | - | cor=−0.01; p=0.956 | - | cor=0.03; p=0.872 | - |
|  | STAT6 | - | - | - | - | - | - | **-** | - | - | **-** | cor=0.14; p=0.461 |
| CD4 T cells | MSGB | cor=0.05; p=0.796 | cor=0.86; p=0.657 | cor=−0.04; p=0.832 | cor=−0.03; p=0.867 | cor=−0.07; p=0.733 | cor=0.11; p=0.563 | cor=0.04; p=0.853 | cor=0.19; p=0.344 | cor=−0.18; p=0.369 | cor=−0.05; p=0.807 | cor=−0.03; p=0.876 |
|  | Prot Phos | cor=0.03; p=0.885 | cor=−0.05; p=0.778 | cor=−0.14; p=0.444 | cor=−0.28; p=0.119 | cor=−0.22; p=0.254 | cor=−0.09; p=0.626 | cor=−0.02; p=0.915 | cor=−0.16; p=0.402 | cor=−0.27; p=0.171 | cor=−0.09; p=0.628 | cor=−0.08; p=0.691 |
|  | Prot reg phos | cor=−0.29; p=0.130 | cor=0.03; p=0.883 | cor=0.04; p=0.827 | cor=−0.14; p=0.436 | cor=−0.28; p=0.135 | cor=−0.03; p=0.883 | cor=0.17; p=0.380 | cor=−0.18; p=0.355 | cor=−0.06; p=0.761 | cor=0.25; p=0.189 | cor=0.03; p=0.858 |
|  | STAT1_4 | - | - | - | - | - | - | cor=0.22; p=0.269 | - | cor=0.17; p=0.383 | - | - |
|  | STAT3_5 | - | - | - | - | - | - | - | cor=0.28; p=0.148 | - | cor=0.03; p=0.878 | - |
|  | STAT6 | - | - | - | - | - | - | **-** | - | - | **-** | cor=0.32; p=0.089 |
| CD8 T cells | MSGB | cor=−0.11; p=0.575 | **cor=−0.43; p=0.018** | cor=−0.21; p=0.258 | cor=−0.05; p=0.776 | cor=−0.07; p=0.722 | cor=−0.22; p=0.254 | cor=−0.26; p=0.166 | cor=0.11; p=0.578 | cor=−0.19; p=0.333 | cor=−0.14; p=0.458 | cor=−0.17; p=0.377 |
|  | Prot Phos | cor=0.10; p=0.586 | cor=−0.32; p=0.088 | cor=−0.18; p=0.334 | cor=−0.25; p=0.172 | cor=−0.21; p=0.253 | cor=−0.18; p=0.359 | cor=−0.36; p=0.055 | cor=−0.23; p=0.220 | cor=−0.29; p=0.126 | cor=−0.22; p=0.248 | cor=−0.20; p=0.313 |
|  | Prot reg phos | cor=−0.24; p=0.194 | cor=−0.27; p=0.148 | cor=−0.05; p=0.778 | cor=−0.06; p=0.737 | cor=−0.22; p=0.234 | cor=−0.04; p=0.841 | cor=−0.05; p=0.799 | cor=−0.03; p=0.883 | cor=−0.04; p=0.838 | cor=0.18; p=0.334 | cor=−0.01; p=0.943 |
|  | STAT1_4 | - | - | - | - | - | - | cor=0.09; p=0.649 | - | cor=0.16; p=0.418 | - | - |
|  | STAT3_5 | - | - | - | - | - | - | - | cor=0.12; p=0.548 | - | cor=−0.07; p=0.697 |  |
|  | STAT6 | - | - | - | - | - | - | **-** | - | **-** | **-** | cor=0.03; p=0.880 |
| NK cells | MSGB | cor=−0.25; p=0.217 | cor=0.02; p=0.929 | cor=0.28; p=0.169 | cor=−0.15; p=0.461 | cor=0.21; p=0.296 | cor=0.03; p=8.81e−01 | cor=−0.24; p=0.266 | cor=−0.36; p=0.086 | cor=−0.16; p=0.466 | cor=−0.27; p=0.197 | cor=0.03; p=0.900 |
|  | Prot Phos | cor=0.001; p=0.995 | cor=−0.11; p=0.607 | cor=0.05; p=0.817 | cor=−0.29; p=0.143 | cor=−0.15; p=0.463 | cor=−0.04; p=0.831 | cor=−0.16; p=0.451 | **cor=−0.53; p=0.008** | cor=−0.10; p=0.658 | cor=−0.15; p=0.487 | cor=0.02; p=0.934 |
|  | Prot reg phos | cor=−0.006; p=0.976 | cor=0.27; p=0.184 | **cor=0.44; p=0.027** | cor=−0.08; p=0.678 | cor=0.19; p=0.358 | cor=0.27; p=0.180 | cor=−0.17; p=0.422 | cor=0.03; p=0.897 | cor=0.06; p=0.801 | cor=0.03; p=0.894 | cor=0.21; p=0.321 |
|  | STAT1_4 | - | - | - | - | - | - | cor=−0.12; p=0.565 | - | cor=−0.01; p=0.953 | - | - |
|  | STAT3_5 | - | - | - | - | - | - | - | cor=−0.12; p=0.565 | - | **cor=−0.49; p=0.014** | - |
|  | STAT6 | **-** | - | - | - | **-** | - | **-** | - | **-** | - | cor=0.11; p=0.595 |

Correlation between levels of phosphorylated proteins after *in vitro* stimulation and the MSGB (MS genetic burden), MSPBphos (pathway burden of protein phosphorylation ontological family) or MSPBregphos (pathway burden of regulation of protein phosphorylation ontological family) in each cell type analyzed. Cor: Spearman coefficient; p: p-values. Significant correlations are highlighted in bold.

| Monocytes | MSGB | cor=0.11; p=0.543 | cor=−0.04; p=0.819 | cor=0.12; p=0.523 | cor=−0.004; p=0.982 | cor=−0.25; p=0.170 | cor=−0.07; p=0.720 | cor=−0.04; p=0.828 | cor=0.11; p=0.565 | cor=−0.17; p=0.374 | cor=−0.15; p=0.438 | cor=−0.006; p=0.974 |
| --- | --- | --- | --- | --- | --- | --- | --- | --- | --- | --- | --- | --- |
|  | Prot Phos | cor=0.22; p=0.232 | cor=−0.06; p=0.736 | cor=−0.004; p=0.982 | cor=−0.06; p=0.732 | cor=−0.24; p=0.200 | cor=−0.29; p=0.116 | cor=−0.162; p=0.421 | cor=−0.310; p=0.101 | **cor=−0.37; p=0.043** | cor=−0.23; p=0.237 | cor=−0.17; p=0.377 |
|  | Prot reg phos | cor=0.17; p=0.360 | cor=0.03; p=0.878 | cor=0.13; p=0.497 | cor=0.07; p=0.713 | cor=−0.15; p=0.415 | cor=0.09; p=0.615 | cor=0.04; p=0.839 | cor=0.09; p=0.639 | cor=−0.09; p=0.618 | cor=0.24; p=0.212 | cor=0.13; p=0.490 |
|  | STAT1_4 | - | - | - | - | - | - | cor=0.09; p=0.636 | - | cor=0.07; p=0.694 | - | - |
|  | STAT3_5 | - | - | - | - | - | - | - | cor=−0.24; p=0.201 | - | cor=−0.25; p=0.197 | - |
|  | STAT6 | - | - | **-** | - | - | - | **-** | **-** | - | **-** | cor=0.13; p=0.486 |
