# Supplementary material for: Aberrant STAT phosphorylation signaling in peripheral blood mononuclear cells from multiple sclerosis patients
Source: J Neuroinflammation. 2018 Mar 7;15:72. doi: 10.1186/s12974-018-1105-9 (PMC5840794; doi:10.1186/s12974-018-1105-9)
Supplement: Supplementary file 10 — Table S9. Correlation between STAT phosphorylation and HLA-E, HLA-ABC, and HLA-DR expression in different cell populations. Correlation between levels of p-STAT1, p-STAT3, p-STAT4, p-STAT5, and p-STAT6 proteins and HLA-E, HLA-ABC, and HLA-DR expression after IFN-α or IFN-γ stimulation. Cor: Spearman coefficient; p: p values. Significant correlations are highlighted in bold. (DOCX 15 kb) [file 12974_2018_1105_MOESM10_ESM.docx]

Table S9. Correlation between STAT phosphorylation and HLA-E, HLA-ABC and HLA-DR expression in different cell populations

| **p-STAT1** | **HLA-E (IFNa)** | **HLA-E**  **(IFNg)** | **HLA-ABC (IFNa)** | **HLA-ABC (IFNg)** | **HLA-DR (IFNa)** | **HLA-DR (IFNg)** |
| --- | --- | --- | --- | --- | --- | --- |
| **B cells** | cor=3.25e−01; p=1.31e−01 | cor=3.49e−01; p=1.03e−01 | cor=2.14e−01; p=3.26e−01 | cor=3.64e−01; p=8.74e−02 | cor=3.58e−01; p=9.37e−02 | **cor=4.54e−01; p=2.94e−02** |
| **CD4 T cells** | cor=−3.41e−02; p=8.77e−01 | cor=2.25e−01; p=3.01e−01 | cor=−1.05e−01; p=6.33e−01 | cor=1.84e−01; p=4.00e−01 | cor=3.70e−01; p=8.26e−02 | cor=2.84e−01; p=1.90e−01 |
| **CD8 T cells** | cor=1.38e−01; p=5.27e−01 | cor=1.26e−01; p=5.68e−01 | cor=2.16e−01; p=3.20e−01 | cor=1.55e−01; p=4.81e−01 | cor=1.17e−01; p=5.96e−01 | cor=2.21e−02; p=9.20e−01 |
| **NK cells** | cor=−2.47e−01; p=2.55e−01 | cor=−2.80e−01; p=1.96e−01 | cor=−2.74e−01; p=2.06e−01 | cor=−4.01e−01; p=5.80e−02 | cor=−1.48e−02; p=9.48e−01 | cor=1.98e−03; p=9.93e−01 |
| **Monocytes** | **cor=4.31e−01; p=4.13e−02** | cor=3.74e−01; p=7.91e−02 | **cor=4.43e−01; p=3.56e−02** | **cor=5.25e−01; p=1.00e−02** | **cor=6.04e−01; p=2.78e−03** | **cor=5.16e−01; p=1.17e−02** |
| **p-STAT3** |  |  |  |  |  |  |
| **B cells** | cor=−1.46e−02; p=9.47e−01 | cor=−1.44e−01; p=5.13e−01 | cor=−2.42e−02; p=9.13e−01 | cor=−2.58e−01; p=2.35e−01 | cor=−9.24e−02; p=6.75e−01 | cor=−2.15e−01; p=3.25e−01 |
| **CD4 T cells** | cor=3.70e−01; p=8.24e−02 | cor=1.61e−01; p=4.62e−01 | cor=3.27e−01; p=1.28e−01 | cor=−1.01e−01; p=6.45e−01 | cor=5.69e−02; p=7.97e−01 | cor=−2.38e−01; p=2.74e−01 |
| **CD8 T cells** | cor=3.90e−02; p=8.60e−01 | cor=−1.49e−01; p=4.98e−01 | cor=2.66e−01; p=2.20e−01 | cor=−2.49e−01; p=2.52e−01 | cor=−1.26e−01; p=5.66e−01 | **cor=−4.69e−01; p=2.40e−02** |
| **NK cells** | cor=−2.68e−01; p=2.16e−01 | **cor=−5.53e−01; p=6.17e−03** | cor=−2.89e−01; p=1.81e−01 | **cor=−7.15e−01; p=1.26e−04** | cor=−5.98e−02; p=7.86e−01 | cor=−5.44e−02; p=8.05e−01 |
| **Monocytes** | **cor=4.24e−01; p=4.50e−02** | cor=1.03e−01; p=6.40e−01 | **cor=5.18e−01; p=1.24e−02** | cor=3.80e−01; p=7.42e−02 | **cor=4.69e−01; p=2.51e−02** | cor=3.66e−01; p=8.69e−02 |
| **p-STAT4** |  |  |  |  |  |  |
| **B cells** | cor=−3.81e−01; p=7.33e−02 | cor=−2.77e−01; p=2.01e−01 | **cor=−4.44e−01; p=3.37e−02** | **cor=−4.72e−01; p=2.29e−02** | **cor=−4.74e−01; p=2.22e−02** | **cor=−4.35e−01; p=3.82e−02** |
| **CD4 T cells** | cor=4.99e−02; p=8.21e−01 | cor=−9.39e−02; p=6.69e−01 | cor=−3.90e−02; p=8.60e−01 | **cor=−4.68e−01; p=2.54e−02** | cor=−2.37e−02; p=9.14e−01 | cor=−3.26e−01; p=1.29e−01 |
| **CD8 T cells** | cor=1.79e−01; p=4.12e−01 | cor=−1.13e−01; p=6.07e−01 | cor=2.17e−01; p=3.18e−01 | cor=−3.90e−01; p=6.62e−02 | cor=4.70e−02; p=8.32e−01 | cor=−2.58e−01; p=2.35e−01 |
| **NK cells** | cor=3.42e−01; p=1.11e−01 | **cor=−4.63e−01; p=2.72e−02** | cor=1.09e−01; p=6.20e−01 | **cor=−7.07e−01; p=2.43e−04** | cor=−2.15e−01; p=3.22e−01 | cor=−1.64e−01; p=4.53e−01 |
| **Monocytes** | **cor=4.23e−01; p=4.55e−02** | cor=−3.51e−02; p=8.74e−01 | **cor=6.17e−01; p=2.14e−03** | cor=2.11e−01; p=3.34e−01 | cor=3.10e−01; p=1.49e−01 | cor=3.85e−01; p=6.97e−02 |
| **p-STAT5** |  |  |  |  |  |  |
| **B cells** | cor=2.71e−01; p=2.11e−01 | **cor=4.43e−01; p=3.43e−02** | cor=1.13e−01; p=6.09e−01 | **cor=4.59e−01; p=2.77e−02** | cor=6.92e−02; p=7.54e−01 | cor=−7.92e−02; p=7.19e−01 |
| **CD4 T cells** | cor=9.52e−02; p=6.66e−01 | cor=9.55e−02; p=6.65e−01 | cor=−3.95e−02; p=8.58e−01 | cor=2.49e−01; p=2.51e−01 | cor=3.69e−01; p=8.29e−02 | cor=−9.40e−03; p=9.66e−01 |
| **CD8 T cells** | cor=1.18e−01; p=5.92e−01 | cor=1.71e−01; p=4.35e−01 | cor=2.21e−01; p=3.09e−01 | **cor=4.93e−01; p=1.69e−02** | cor=1.80e−01; p=4.10e−01 | cor=1.25e−01; p=5.71e−01 |
| **NK cells** | cor=−1.95e−01; p=3.72e−01 | cor=−2.48e−01; p=2.54e−01 | cor=−2.61e−01; p=2.28e−01 | cor=−3.29e−01; p=1.26e−01 | cor=−1.48e−02; p=9.48e−01 | cor=3.46e−02; p=8.75e−01 |
| **Monocytes** | **cor=4.26e−01; p=4.39e−02** | cor=3.90e−01; p=6.65e−02 | **cor=5.22e−01; p=1.17e−02** | **cor=5.68e−01; p=5.40e−03** | **cor=5.30e−01; p=1.03e−02** | cor=2.03e−01; p=3.52e−01 |
| **p-STAT6** |  |  |  |  |  |  |
| **B cells** | cor=8.75e−02; p=6.91e−01 | cor=7.23e−02; p=7.43e−01 | cor=−1.63e−01; p=4.59e−01 | cor=−1.06e−01; p=6.31e−01 | cor=−5.04e−02; p=8.19e−01 | cor=−2.57e−01; p=2.37e−01 |
| **CD4 T cells** | cor=1.99e−01; p=3.63e−01 | cor=4.01e−02; p=8.56e−01 | cor=−1.12e−01; p=6.12e−01 | cor=−2.11e−01; p=3.35e−01 | cor=5.93e−02; p=7.88e−01 | cor=−2.31e−01; p=2.89e−01 |
| **CD8 T cells** | cor=3.01e−01; p=1.62e−01 | cor=−2.52e−01; p=2.46e−01 | cor=2.79e−01; p=1.97e−01 | cor=−2.76e−01; p=2.02e−01 | cor=8.06e−02; p=7.15e−01 | cor=−2.35e−01; p=2.80e−01 |
| **NK cells** | cor=1.61e−01; p=4.64e−01 | **cor=−6.12e−01; p=1.91e−03** | cor=9.88e−04; p=9.96e−01 | **cor=−7.40e−01; p=5.43e−05** | cor=−1.54e−01; p=4.82e−01 | cor=−8.16e−02; p=7.11e−01 |
| **Monocytes** | cor=3.81e−01; p=7.34e−02 | cor=−3.16e−02; p=8.87e−01 | **cor=5.84e−01; p=4.05e−03** | cor=1.18e−01; p=5.92e−01 | **cor=4.60e−01; p=2.83e−02** | cor=9.29e−02; p=6.72e−01 |

Correlation between levels of p-STAT1, p-STAT3, p-STAT4, p-STAT5 and p-STAT6 proteins and HLA-E, HLA-ABC and HLA-DR expression after IFN-α or IFN-γ stimulation. Cor: Spearman coefficient; p: p-values. Significant correlations are highlighted in bold.
